# Functional and clinical validation of tsRNA-defined molecular subtypes guides precision therapy in gastric cancer

**DOI:** 10.3389/fimmu.2025.1684113

**Published:** 2025-11-03

**Authors:** Ye Tian, Xin Hu, Yuan Liu, Wenqi Wu, Yanxin Yao, Huahuan Liu, Wei Wang, Hongji Dai, Yubei Huang, Changyu Sun, Yan Cui, Zun Li, Xiangnan Zhang, Liqing Jia, Fubing Wang, Fengju Song, Kexin Chen, Yuan Pan, Ben Liu

**Affiliations:** ^1^ Department of Epidemiology and Biostatistics, Key Laboratory of Molecular Cancer Epidemiology, Ministry of Education, National Clinical Research Center for Cancer, Tianjin Medical University Cancer Institute and Hospital, Tianjin Medical University, Tianjin, China; ^2^ Key Laboratory of Prevention and Control of Human Major Diseases, Ministry of Education, National Clinical Research Center for Cancer, Tianjin Medical University Cancer Institute and Hospital, Tianjin Medical University, Tianjin, China; ^3^ Department of Gastric Surgery, Tianjin Medical University Cancer Institute & Hospital, National Clinical Research Center for Cancer, Tianjin, China; ^4^ Tianjin Key Laboratory of Digestive Cancer, Tianjin, China; ^5^ Tianjin’s Clinical Research Center for Cancer, Tianjin, China; ^6^ Center for Single-Cell Omics and Tumor Liquid Biopsy, Zhongnan Hospital of Wuhan University, Wuhan, China; ^7^ Department of Laboratory Medicine, Zhongnan Hospital of Wuhan University, Wuhan, China; ^8^ Wuhan Research Center for Infectious Diseases and Cancer, Chinese Academy of Medical Sciences, Wuhan, China

**Keywords:** gastric cancer, tsRNA subtype, machine learning, prognostic model, organoid

## Abstract

**Introduction:**

Gastric cancer (GC) is a highly heterogeneous malignancy with poor prognosis, underscoring the urgent need for reliable biomarkers to guide precise stratification and therapy. Transfer RNA-derived small RNAs (tsRNAs) have emerged as potential key regulators in cancer, yet their systematic role in defining GC subtypes remains unexplored.

**Methods:**

We profiled tsRNA expression in GC using transcriptomic data from TCGA and GEO databases. Unsupervised consensus clustering identified tsRNA-based subtypes. A prognostic model was constructed using machine learning algorithms and validated across multiple cohorts. The functional role of a key tsRNA, tsRNA-Asp-3-0024, was investigated through Pandora-seq, qRT-PCR, and *in vitro* and organoid-based assays.

**Results:**

Three distinct tsRNA-mediated subtypes (Stromal_H, Stromal_L, Stromal_M) were identified, exhibiting significant differences in stromal activity, tumor microenvironment, and clinical outcomes. The Stromal_H subtype demonstrated the poorest prognosis, characterized by an immunosuppressive microenvironment and dysregulated DNA repair pathways. A random survival forest (RSF)-based prognostic signature (GCtsRNAscore) effectively stratified patients into high- and low-risk groups, with high-risk patients showing increased sensitivity to targeted therapies (axitinib, bexarotene, dasatinib) and low-risk patients benefiting more from immunotherapy. Furthermore, tsRNA-Asp-3-0024 was significantly upregulated in GC tissues and cell lines, where it promoted proliferation and inhibited apoptosis.

**Discussion:**

Our study establishes tsRNAs as powerful biomarkers for molecular subtyping and prognostic prediction in GC. The tsRNA-defined subtypes and GCtsRNAscore model provide a novel framework for personalized treatment strategies. The functional characterization of tsRNA-Asp-3-0024 highlights its potential as both a therapeutic target and a prognostic indicator, paving the way for tsRNA-based precision medicine in GC.

## Graphical Abstract

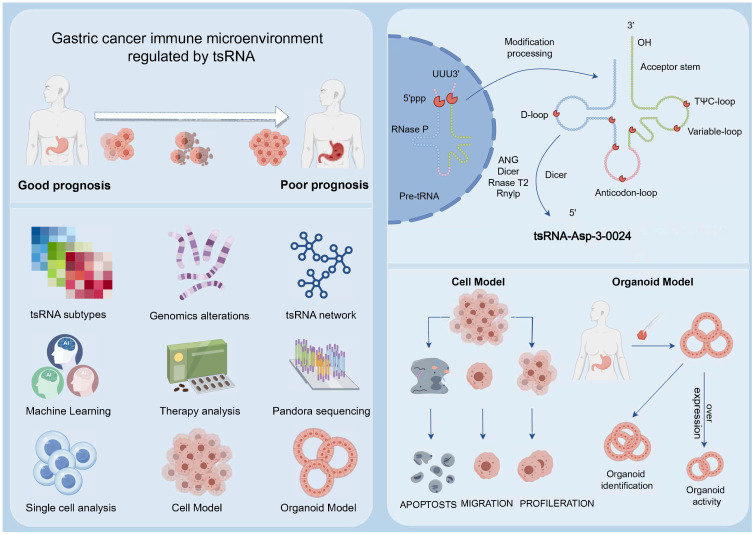

## 1 Introduction

Gastric cancer (GC) remains a significant global health challenge, ranking among the most common malignancies and accounting for over 780,000 deaths annually (1). Incidence rates are notably higher in men than women, and there is a concerning increase in younger patients, likely related to changes in lifestyle and environmental exposure (2). The persistently high mortality rate is mainly due to late-stage diagnosis and metastasis, which limit therapeutic options and result in poor prognosis (3).

Current diagnostic practices, such as upper gastrointestinal endoscopy, are invasive and heavily dependent on operator expertise, leading to discomfort and variable patient detection rates (4, 5). Common serum biomarkers, including carcinoembryonic antigen (CEA), carbohydrate antigen 19-9 (CA19-9), and CA72-4, demonstrate limited sensitivity and specificity for GC detection (6). Despite advances in immunotherapy and targeted treatments, including trastuzumab, ramucirumab, and immune checkpoint inhibitors, clinical responses vary, and many patients fail to benefit from these approaches (7, 8). Despite the remarkable progress achieved through immunization and targeted therapies in treating gastric cancer, individual responses to these approaches can be variable. Therefore, identifying new therapeutic targets and novel biomarkers for early screening is crucial to enhancing the prognosis of patients with gastric cancer.

Noncoding RNAs (ncRNAs) have emerged as important regulators in cancer biology and are actively being explored for their diagnostic and prognostic potential (6). Among these, transfer RNA-derived small RNAs (tsRNAs) are a class of noncoding RNAs produced from precursor or mature tRNAs under various cellular stress conditions (9–12). TsRNAs-including tRNA halves (tiRNAs) and tRNA-derived fragments (tRFs)-have been implicated in gene regulation, mRNA stability, translation, and epigenetic modulation (13–19). Many tsRNAs are stable and detectable in biological fluids, supporting their potential as non-invasive biomarkers (20, 21).

Emerging evidence reveals that select tRNA-derived fragments (tRFs/tiRNAs) exhibit dysregulation in stress responses and oncogenesis (22). Their remarkable stability in circulation, particularly within blood-derived extracellular vesicles [e.g., MCF7 breast cancer models (23)], establishes them as potent signaling molecules with biomarker potential. Mounting clinical evidence demonstrates aberrant tRF expression across malignancies (24). Critically, their detectability in biofluids and functional implications position them as non-invasive diagnostic indicators. For instance, diminished tiRNA-5034-GluTTC-2 expression predicts accelerated progression in gastric cancer patients, supporting its utility as a prognostic biomarker (25). Given the conserved prevalence of tRFs in biological systems (26), these molecules represent promising translational targets for enhancing GC outcomes through early detection and precision intervention.

PANDORA-seq (27) revolutionized small RNA profiling by uncovering a vast repertoire of post-transcriptionally modified sncRNAs, predominantly tRNA-derived (tsRNA) and rRNA-derived (rsRNA) fragments. Previously obscured in conventional sequencing, these sncRNAs exhibit striking tissue-specific expression across murine organs (brain, liver, spleen, sperm) and cell-type-specific dynamics in embryonic stem cells (ESCs) versus HeLa cells. This technological advance provides the foundation for identifying clinically actionable sncRNA biomarkers in human cancers.

These challenges underscore the urgent need for novel biomarkers to improve early detection, prognostic stratification, and personalized therapy in gastric cancer. To address these gaps, this study aims to systematically characterize tsRNA profiles through integrated multi-omics approaches and experimental validation.

## 2 Materials and methods

### 2.1 Single-cell RNA sequencing data

The scRNA-seq dataset (GSE167297), derived from five gastric cancer samples, was processed and analyzed using the Seurat package. To ensure quality, cells were retained only if they contained between 300 and 10,000 transcripts, expressed genes detected in at least three cells, and exhibited mitochondrial read percentages below 5%. All cells were grouped by applying a clustering resolution parameter of 0.5. Major cell types were identified based on established reference markers from literature or the CellMarker database.

### 2.2 Genomic mutation analysis

Somatic mutation data were retrieved from the TCGA database. Significant mutated genes were detected using the “maftools” R package. We retrieved the mutational signature of liver cancer patients and compared them with the mutation database (COSMIC V2) using the cosine similarity method (https://cancer.sanger.ac.uk/cosmic/).

### 2.3 Geneset variation analysis

To examine biological pathway disparities among tsRNA subtypes, we used Gene Set Variation Analysis (GSVA) to leverage the eponymous R package. This non-parametric, unsupervised approach is widely adopted for assessing pathway activity variation and biological process alterations within expression datasets (28). “Hallmark gene sets” were retrieved from the MSigDB database for the analysis. A threshold of P < 0.05 defined statistical significance.

### 2.4 Immunophenoscore

We employed Immunophenoscore (IPS), a superior molecular marker for immune response, to characterize intratumoral immune landscapes and cancer antigenomes. This scoring system integrates features derived from four immune-related gene clusters: major histocompatibility complex (MHC) molecules, immunomodulators/checkpoints, effector cells, and suppressor cells. Elevated IPS values correlate with enhanced immunotherapeutic efficacy (29).

### 2.5 Tumor mutational burden and microsatellite instability status

The data on TMB were obtained from a previous study (30). Microsatellite instability (MSI) status was stratified into three categories: microsatellite stable (MSS), MSI-low (MSI-L; one unstable marker), and MSI-high (MSI-H; >2 unstable markers). Corresponding classification data were sourced from the UCSC Xena database.

### 2.6 Copy number variation

We assessed copy number variation (CNV) within tsRNA genomic regions. Significant CNV segments (alteration frequency > 0.3) in gastric cancer (GC) were further identified. Visualization of subtype-specific CNV patterns was achieved using the *ComplexHeatmap* R package: a waterfall plot illustrated significant CNV segments across three tsRNA subtypes, while a heatmap depicted corresponding alteration frequencies.

### 2.7 Data collection and preprocessing

The study workflow is shown in **Supplementary Figure S1**. This study employed publicly available gene expression data from TCGA and the Gene Expression Omnibus (GEO) database. Corresponding clinical data of TCGA gastric cancer were extracted from the UCSC Xena web data resource (https://xenabrowser.net/datapages/). For further evaluation, patients who did not have survival information available were excluded from the analysis. Utilizing TCGA data (https://portal.gdc.cancer.gov/), we calculated fragments per kilobase per million (FPKM) values. These FPKM metrics, sourced from the TCGA program, were converted to transcripts per kilobase million (TPM). For microarray datasets from Affymetrix^®^ in the GEO database, raw “CEL” files were acquired and preprocessed via R’s affy package, implementing robust multiarray averaging normalization. Normalized matrix files from other GEO platforms were directly retrieved. RNA-seq cohort data were processed using the GENCODE v22 resource (http://www.gencodegenes.org/). Five gastric cancer cohorts met the inclusion criteria: GSE13861 (N = 64), GSE15459 (N = 192), GSE26901 (N = 109), GSE34942 (N = 56), and TCGA-STAD (N = 349). TCGA and GEO mRNA expression datasets underwent independent normalization. Somatic mutation profiles originated from UCSC Xena, while gastric cancer copy number alterations were derived from Broad GDAC Firehouse (http://gdac.broadinstitute.org/). tsRNA expression matrices and metadata were obtained from the tsRFun database (27).

### 2.8 Consensus cluster of GC-specific tsRNAs

TsRNAs detected in at least 70% of the samples with a mean RPM value >1 were used for data analysis. Subsequently, we establish the designation of gastric cancer-specific tsRNAs by identifying tsRNAs that exhibit differential expression patterns between cancer and para-cancer. To delineate heterogeneous patterns in tsRNA transcriptional regulation, we performed unsupervised consensus clustering based on expression profiles of 80 gastric cancer (GC)-specific tsRNAs. This algorithm simultaneously quantified optimal cluster quantity and partition stability, thus establishing molecular subtypes for downstream analysis. For the steps mentioned above, we utilized the ConsensusClusterPlus package, which facilitated the execution of the consensus clustering analysis. To ensure robustness and stability in the classification, we performed 100 repetitions, allowing for a comprehensive assessment of the cluster assignments and enhancing the results’ reliability.

### 2.9 Estimation of TME cell infiltration between tsRNA subtypes

The relative abundance of each cell infiltration in the GC TME was determined using the single-sample gene set enrichment analysis (ssGSEA) algorithm. A previous study identified biomarker genes specific to 28 types of immune cells. Among the 28 immune cell subtypes are MDSCs, activated dendritic cells, macrophages, natural killer T cells, and regulatory T cells (29, 31). Using ssGSEA enrichment scores, we could estimate the relative abundance of each TME-infiltrating cell within each sample. ESTIMATER was used to calculate the Stromal Score in this study. A Stromal Score was calculated based on the TPM values obtained from RNA-seq data. This score was used to stratify tsRNA clusters further.

### 2.10 Multiple machine learning algorithms were utilized to construct tsRNA-related prognostic model

A scoring system was developed for each patient to determine the regulatory extent of tsRNAs expressed within their tumors. The term “tsRNA score” (RS) was assigned as the name for this specific tsRNA signature. The following are the steps taken to establish the tsRNA signature:

Prognostic associations of tsRNA-related genes were determined via univariate Cox regression. Subsequent machine learning analyses exclusively incorporated genes demonstrating statistical significance (*P* < 0.005). We utilized a total of 74 combinations of machine-learning algorithms to develop a prognostic model, incorporating ten specific algorithms: Lasso, Enet, plsRcox, CoxBoost, StepCox, GBM, Ridge, RSF, survival-SVM, and SuperPC. To ensure model robustness and mitigate overfitting, the training process employed 10-fold cross-validation repeated 5 times for hyperparameter optimization. Model performance was evaluated using the concordance index (C-index). Each of these algorithms can calculate the RS score in GC patients. Employing the R survminer package, patients underwent stratification into high- and low-recurrence score (RS) groups based on optimal cutoff values. The Kaplan-Meier method evaluated both high- and low-RS patients for overall survival (OS). This study indicated statistical significance at *P* < 0.05 using the Log-rank test.

### 2.11 Predict chemotherapeutic response

We employed the R package “pRRophetic” (32) to forecast chemotherapeutic response in gastric cancer (GC) patients. Sample half-maximal inhibitory concentration (IC50) values were derived via ridge regression, with prediction accuracy evaluated through 10-fold cross-validation on the GDSC training set (33).

### 2.12 Pandora sequencing

In order to analyze the differential expression of tsRNA in cancerous and adjacent non-cancerous tissues, Pandora sequencing (Panoramic RNA Display by Overcoming RNA modification Aborted sequencing)was performed according to previous methods (27, 34) on tissue samples obtained from three patients diagnosed with gastric cancer. The study included three cancer tissues and three adjacent non-cancerous tissues.

### 2.13 Clinical samples and cell lines

The Institutional Review Board of Tianjin Medical University Cancer Institute and Hospital granted ethical approval for this investigation. Clinical gastric cancer specimens were procured from surgical resections conducted within the Department of Gastrointestinal Oncology at the aforementioned institution. Human gastric cancer cell lines HGC-27, AGS, MKN45, and normal human gastric mucosa cells GES were obtained from the Chinese Academy of Biological Sciences (Shanghai) cell bank. SGC-7901 cell lines were purchased from GENECHEM. All five cell lines were cultured in RPMI 1640 medium with 10% fetal bovine serum at 37°C with 5% CO2 in proper humidity. Authentication via short tandem repeat (STR) profiling confirmed cellular identity, and comprehensive mycoplasma screening yielded negative results across all lines.

### 2.14 Organoid construction and characterization

Fresh tissues of human gastric cancer and adjacent normal tissues were obtained by surgical excision of the specimens. Gastric cancer tissues were rinsed with antibiotic-containing PBS, then the fresh tissues were minced with scissors, placed in a 15-cm centrifuge tube containing tissue digestive solution, and digested at 37°C for 10–30 min. A portion of the digestive solution containing the tissues was aspirated at any time during the digestion process for observation under a microscope, and the digestion process was terminated when the majority of the digested tissues were found to be cellular agglomerates. The digested tissue fragments were filtered through a 70 μm filter, and the filtrate was collected and centrifuged at 4°C for 5 min at 250 g. If the precipitate was red blood cells, 2 ml of lysate was added for lysis. After centrifugation, the precipitate was washed twice and transferred to a 1.5 ml centrifuge tube. The cell precipitate was collected and resuspended with organoid-specific matrix gel(CORNING, USA), and the cell-matrix gel mixture was quickly spread on a pre-warmed 24-well plate. The 24-well plates were placed in the incubator and left to stand for 30 minutes until the matrix gel formed a soft gel, and then covered with an organoid complete medium. Twenty-four hours later, the organoids could be observed under the microscope in clusters. The organoid complete medium was replaced with fresh organoid complete medium every three days, and passaging could be carried out in about 15 days. When the size of the organoids averaged 200 μm, the organoids in the well plates could be collected and fixed with 4% paraformaldehyde and embedded for HE staining to compare with the pathological results of the patient’s source.

### 2.15 RNA isolation and quantitative RT-PCR

Total RNA isolation from cellular and tissue specimens employed Trizol reagent (Thermo Fisher, USA). All samples exhibited OD 260/280 ratios of 1.8-2.0 before storage at -80°C in RNase-free water. RNA integrity and concentration were quantified via NanoDrop 2000 spectrophotometry (Thermo Fisher). For tsRNA-Asp-3–0024 analysis, reverse transcription utilized Bulge-Loop miRNA qRT-PCR Starter Kit (Ribobio, China) with gene-specific stem-loop primers under RT reaction conditions of 42°C for 60 min and 70°C for 10 min. Then, the samples were analyzed with the SYBR Premix Ex Taq (Takara) for qPCR. After adding forward and reverse primers, the reaction was incubated at 95°C for 10 min, 95°C for 10 s, 60°C for 20 s, 70°C for 10 s, and cycling for 40 times. TsRNA expression normalization referenced U6 snRNA, employing Ruibo Biotechnology-designed primers (Guangzhou).

### 2.16 Cell and organoid transfection

For cell transfection, cells were inoculated into petri dishes overnight before transfection. Synthetic stranded inhibitor (tsRNA-Asp-3–0024 inhibitor, 50nM) was purchased from Reebok Biotechnology Ltd. RNA oligonucleotides were transiently transfected with RNAiMAX transfection reagent (Thermo Scientific Dharmacon Inc, USA). For organoid transfection, the organoids were digested with tissue digest at 37°C for 10–30 min centrifuged. The precipitates were washed twice and resuspended in 1.5 ml centrifuge tubes. The transfection complexes were incubated in the organoid suspension for 4 h. Upon completion of the incubation, the cellular precipitates were collected by centrifugation and resuspended with organoid-specific matrix gel, and the cell-matrix gel mixture was rapidly spread in pre-warmed 24-plate and 96-well plates. 96-well plates. The 24- and 96-well plates were placed in an incubator for 30 min until the matrix gel formed a soft gel, and the organoids were covered with complete medium mixed with transfection complexes, respectively. After 12 h, 24 h, 48 h, 72 h, and 96 h of incubation, the growth status was recorded by microscopic photographs. The viability of the organoids was detected, and growth curves were plotted using the 3D Cell Viability Assay Kit (Vazyme, Nanjing), and the absorbance of the medium plus the assay reagent at the time of organoids was used as a background control. Experiments were performed in triplicate.

### 2.17 Cell proliferation, colony formation, and apoptosis assays

Cell proliferative activity was assessed with Cell Counting Kit 8 (CCK8; KeyGEN BioTECH, Jiangsu) per manufacturer’s protocol. HGC-27 or AGS cells (1,500 cells/well) were seeded in 96-well plates. Following 12-72h culture intervals, cultures were supplemented with 10μl CCK-8 solution and underwent 2h incubation at 37°C. Optical density at 450nm was quantified via a microplate reader, using cell-free medium/CCK-8 mixtures as background controls. All assays incorporated technical triplicates.

Following 48-hour inhibitor transfection, cells were subjected to colony formation assays by seeding in 6-well plates (1×10³ cells/well) with 8–10 day incubation. Subsequent processing involved fixation in 4% paraformaldehyde and 0.1% crystal violet staining. Using representative images, the relative colony-forming capacity was quantified through ImageJ analysis (NIH, USA).

For the cell apoptosis assay, the transfected cells (8 × 10^^5^ cells per well) were evenly spread into six-well plates for culture. After the cells were attached to the wall, a line was drawn in the six-well plate with the same strength of the pipette tip of 10 μL, and the degree of healing was observed and photographed under the microscope at 0h, 24h, and 48h after the drawing of the line, respectively. The wound healing percentage was calculated as (wound width at 0 h − wound width at 24 h)/wound width at 0 h.

The cell apoptosis assay was performed by using an Annexin V-fluorescein isothiocyanate (FITC) Apoptosis Detection Kit I (BD Biosciences, USA). Seed the cells to be tested into a 6-well plate or culture dish and culture until the logarithmic growth phase. Remove the culture medium and gently wash the cells twice with pre-chilled PBS. Add an appropriate amount of trypsin (without EDTA) to digest the cells, and once the cells have rounded up, add complete culture medium to terminate digestion. Subject the cell suspension to centrifugation (1000 rpm, 5 min) with subsequent supernatant removal. Resuspend the pellet in ice-cold PBS, perform two sequential washes under identical centrifugation parameters, then reconstitute cells in 1× Binding Buffer at 1×10^6^ cells/mL. Aliquot 100 μL suspension into flow cytometry tubes, introduce 5 μL Annexin V-FITC with gentle vortexing, and incubate protected from light (RT, 15 min); subsequently add 5 μL PI with mixing followed by dark incubation (RT, 5 min). Add 400 μL of 1× Binding Buffer to terminate staining, gently mix, and immediately analyze on the instrument. For organoid transfection, digest the organoids with tissue digestion solution at 37°C for 45 minutes to dissociate cell clusters into single cells. Subject the single-cell suspension to centrifugation (500g, 5 min) with supernatant removal. Resuspend the pellet in 4°C-chilled PBS, followed by two sequential washes under identical centrifugation parameters. The procedure was identical to that used for cell handling.

### 2.18 Statistical analysis

Statistical analyses were performed using R v4.1.1 (freely accessible at https://www.r-project.org) and SPSS 22.0 (IBM, USA) with GraphPad Prism 9.0. Intergroup comparisons utilized: Kruskal-Wallis test for ≥3 groups; Student’s t-test or Mann-Whitney U-test for two groups. Survival prognoses incorporated Kaplan-Meier curves with Log-rank testing (significance threshold *P* < 0.05). Optimal expression-based patient stratification was achieved via survminer-derived cutoff values. Continuous data represent mean ± SD from ≥3 independent experiments. Statistical significance was defined as *P < 0.05, **P < 0.01, ***P < 0.001, ****P < 0.0001 (ns: non-significant), applying two-tailed testing throughout.

## 3 Result

### 3.1 Identification of tsRNA-mediated gastric cancer subtypes

**Figure 1:**
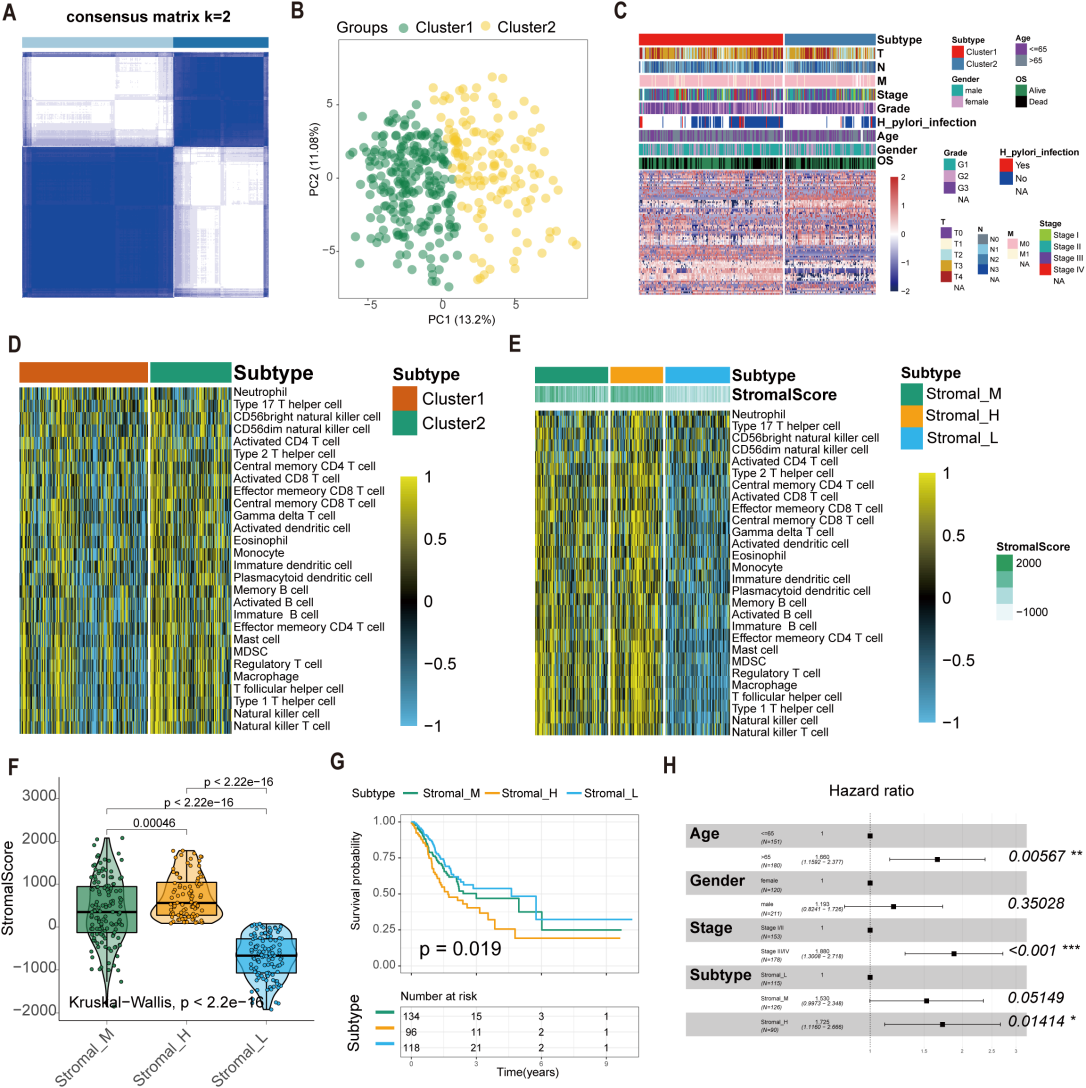
Construction of the tsRNA subtype with distinct immune infiltration and prognosis of GC. **(A)** Cluster optimization derived K = 2 from cumulative distribution function analysis, demonstrating optimal segregation. **(B)** Principal component analysis visualizing GC sample distribution. **(C)** Patient stratification into Cluster1/2 groups with tsRNA expression Z-score normalization. **(D, E)** Single-sample GSEA quantifying relative infiltration of 28 immune cell subpopulations across distinct tsRNA subtypes. The relative infiltration of each cell type was normalized into the Z-score. **(F)** The boxplot showed a statistical difference in Stromal Score between the three tsRNA subtypes (*P* < 2.2e-16). **(G)** Survival analysis of three tsRNA subtypes in the TCGA GC cohort was created using Kaplan-Meier curves. **(H)** Multivariate Cox regression analyses of the association between clinicopathological factors and OS of GC patients in the TCGA cohort. *p < 0.05, **p < 0.01, ***p < 0.001.

We extracted expression profiles of 80 GC-specific tsRNAs from the tsRFun database (**Supplementary Table S1**). Consensus clustering analysis was performed on 349 TCGA GC samples with complete survival information. Cumulative distribution function (CDF) curves indicated optimal stability at k = 2 clusters (**Figures 1A, B**). Accordingly, GC samples were stratified into two primary tsRNA-based clusters: tsRNA-cluster 1 (n = 214) and tsRNA-cluster 2 (n = 135). A heatmap visualizes the overall tsRNA expression patterns across these clusters (**Figure 1C**). Single-sample gene set enrichment analysis (ssGSEA) of immune cell infiltration revealed distinct patterns within tsRNA-cluster 1, suggestive of both “hot” and “cold” tumor immune phenotypes (**Figure 1D**). Given the critical contribution of stromal cells to tumor immune evasion (35), we further investigated the molecular heterogeneity within tsRNA-cluster 1 by analyzing stromal activation scores derived from gene expression profiles. Among the 214 patients in tsRNA-cluster 1, 96 (45%) exhibited high stromal scores, while 118 (55%) had low stromal scores (**Figure 1E**).

This refined analysis delineated three distinct GC subtypes based on the integration of tsRNA expression and stromal activity:

1. Stromal_H: High-stromal score group derived from tsRNA-cluster 1 (n = 96, 45%).
2. Stromal_L: Low-stromal score group derived from tsRNA-cluster 1 (n = 118, 55%).
3. Stromal_M: Representative of tsRNA-cluster 2 (n = 135) (**Figure 1F**).

Survival analysis demonstrated significant overall survival (OS) differences among these subtypes (log-rank *P* = 0.019). Patients with the Stromal_H subtype exhibited the poorest prognosis, while those with the Stromal_L subtype had the most favorable outcomes (**Figure 1G**). Multivariate Cox regression analysis results confirmed the tsRNA-mediated stromal subtype as an independent prognostic factor (*P* = 0.01414) (**Figure 1H**). Meanwhile, we employed the random forest algorithm to map the subtypes and calculated the classification results in two additional datasets. Kaplan-Meier (K-M) analysis was then performed, and the results showed that in GSE15459 (P = 0.018) and GSE84437 (*P* = 0.0079), the p-values for the three subtypes were all less than 0.05. Moreover, the prognostic order was consistent across these datasets, with Stromal_H having the worst prognosis, followed by Stromal_M, and Stromal_L having the best prognosis (**Supplementary Figure S2A, B**).

We have performed a comparative analysis of our subtypes with the TCGA and ACRG subtypes. Using the Fisher exact test, we found that our subtypes show distinct characteristics compared to the TCGA (P = 0.004027) and ACRG (P = 6.19e-09) classifications (**Supplementary Figure S3 A, B**).

### 3.2 Construction of the tsRNA regulatory network in gastric cancer

To elucidate the molecular framework underlying the previously identified tsRNA-mediated gastric cancer (GC) subtypes (Stromal_H, Stromal_M, Stromal_L), we investigated their associated transcriptomic landscapes. Employing Bioconductor’s limma package, we first conducted differential expression analysis on mRNA profiles across the three subtypes (cutoffs: *P* < 0.05 and |log2 fold change| > 0.5). This analysis identified 375 differentially expressed mRNAs (DEmRNAs) (**Supplementary Figure S4**, **Supplementary Table S2**).

**Figure 2:**
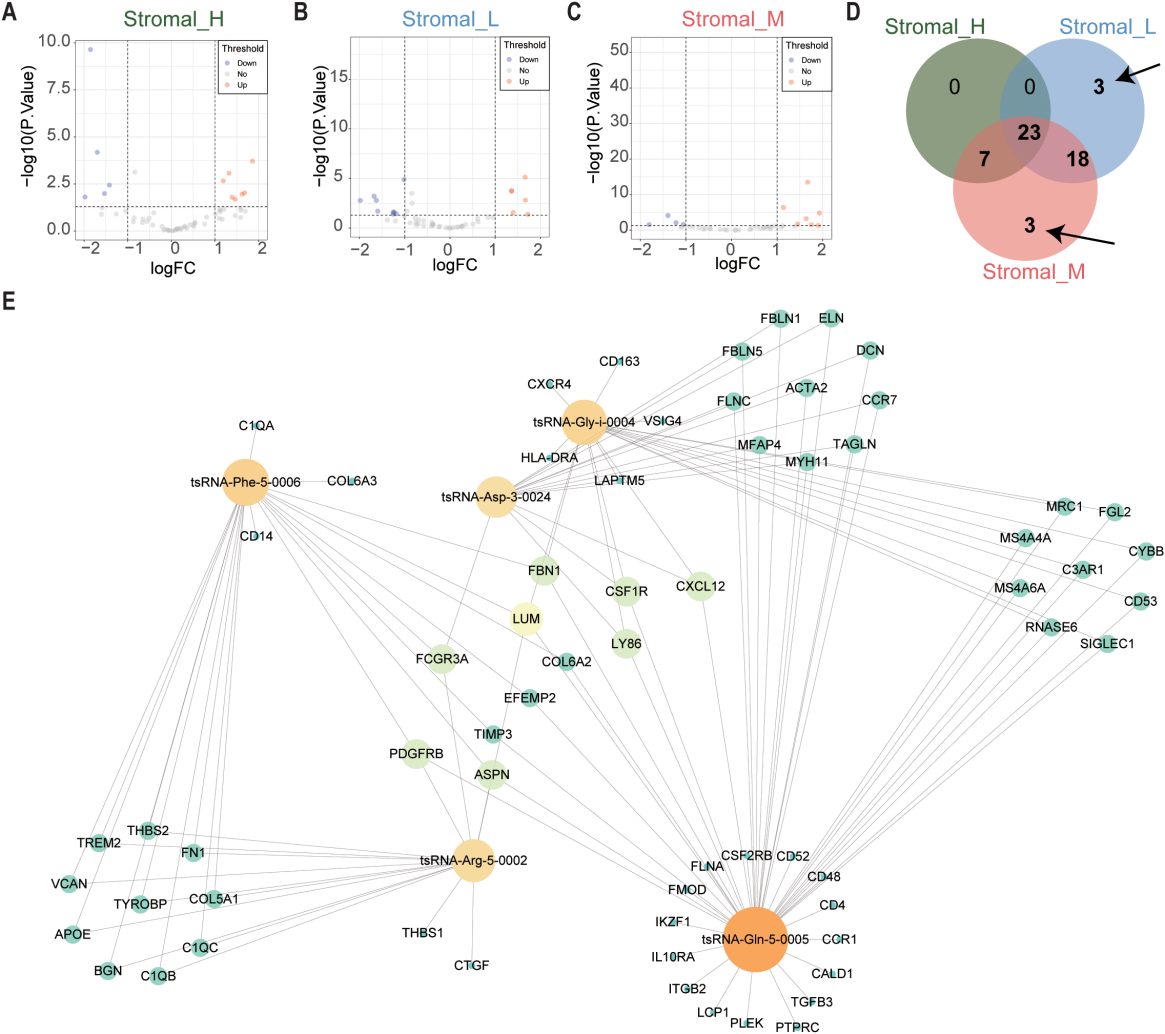
tsRNAs network in GC. **(A)** The volcano plot showed that differentially expressed tsRNAs between Stromal_H and no_Stromal_H subtypes. Each red dot showed an upregulated tsRNA, and each blue dot showed a downregulated tsRNA. **(B)** Volcano plots illustrated differential tsRNA expression profiles between Stromal_L and no_Stromal_L subtypes, where red and blue data points indicate up- and down-regulated tsRNAs respectively **(C)** Stromal_M and no_Stromal_M subtypes, with equivalent color coding denoting analogous expression alterations. **(D)** The veen plot showed that six differentially expressed tsRNAs between the three tsRNA subtypes. **(E)** tsRNA network in GC.

Subsequently, we focused on tsRNA expression differences specific to these subtypes using the same limma framework (cutoffs: *P* < 0.05 and |log2 fold change| > 1). This stringent filtering yielded six differentially expressed tsRNAs (DEtsRNAs) (**Figures 2A–D**), highlighting potential subtype-specific regulatory drivers.

We constructed a protein-protein interaction (PPI) network using the 375 DEmRNAs to integrate these findings and explore potential functional connections. Nodes exhibiting high connectivity (degree centrality > 20) were identified as 67 hub genes (**Supplementary Table S3**), representing central components within this dysregulated transcriptomic network.

Finally, we integrated the expression data of the six DEtsRNAs and the 67 hub DEmRNAs to construct a comprehensive tsRNA regulatory network using Cytoscape software (**Figure 2E**). This integrative network model visualizes potential interactions between subtype-specific tsRNAs and core downstream mRNA targets in gastric cancer, providing a framework for further functional exploration.

### 3.3 Significant differences of biological features and CNV in tsRNA subtypes

**Figure 3:**
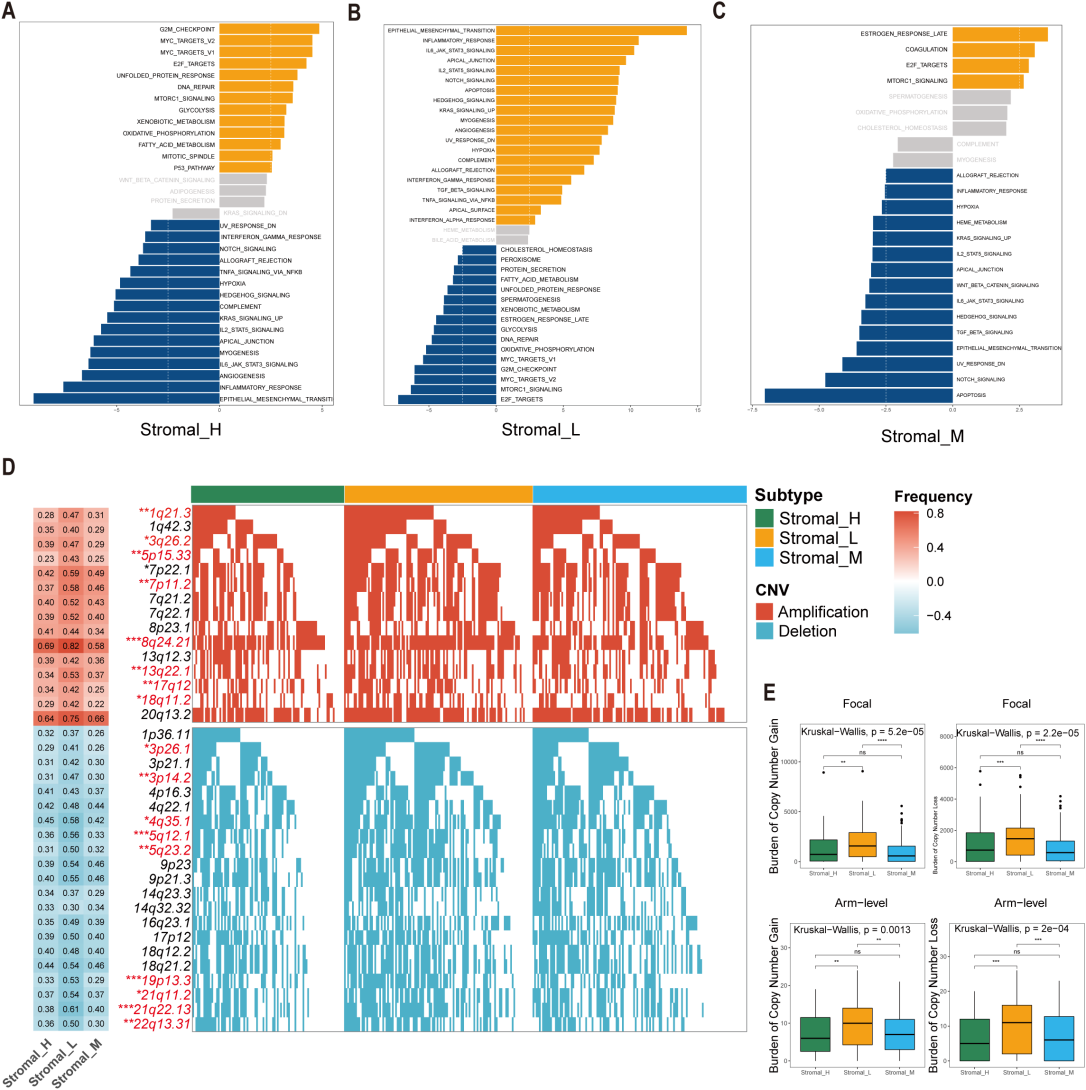
Significant differences of biological features and CNV in tsRNA subtypes. **(A–C)** GSVA analysis depicted differential pathway activation patterns across three tsRNA subtypes. Yellow bars indicate pathways that are significantly activated in the corresponding subtype, while blue bars represent pathways that are significantly inactivated in the corresponding subtype (|t value| > 2.5 and P < 0.05). **(D)** Copy number variation (CNV) profiles delineating subtype-specific gains (upper) and losses (lower). The left heatmap illustrates the frequency of CNV events for specific chromosomal regions, with red indicating amplifications and blue indicating deletions. The intensity of the colors reflects the frequency of CNV events, where darker shades represent higher frequencies. Numerical values within the heatmap denote the specific CNV frequency for each chromosomal region. The right panel presents mutation waterfall plots for each chromosomal segment, depicting the CNV status across the three subtypes, ***P < 0.001, **P < 0.01, *P < 0.05. **(E)** Distribution of focal versus broad-scale CNV alterations among subtypes, with statistical significance denoted as ***P < 0.001, **P < 0.01, *P < 0.05, ns.

The Gene Set Variation Analysis (GSVA) revealed distinct pathway activation patterns among the three stromal subtypes. The Stromal_M subtype exhibited marked enrichment in coagulation and MTORC1 signaling pathways, suggesting potential therapeutic vulnerabilities to pathway-specific inhibitors. Contrary to the findings in the Stromal_L group, the Gene Set Variation Analysis (GSVA) revealed that the G2M checkpoint pathway was upregulated in the Stromal_H group (**Figures 3A–C**).

Comprehensive copy number variations (CNVs) analysis revealed subtype-specific genomic instability patterns. Significant differences emerged in focal amplification events at 1q21.3 (Fisher-test, *P* < 0.01), 3q26.2 (Fisher-test, *P* < 0.05), and 5p15.33 (Fisher-test, *P* < 0.01), as well as deletion hotspots at 3p26.1 (Fisher-test, *P* < 0.05), 3p14.2 (Fisher-test, *P* < 0.01), and 4q35.1 (Fisher-test, *P* < 0.05) (**Figure 3D**). The Stromal_L subtype carried the highest burden of both arm-level gains and losses (**Figure 3E**), as determined by GISTIC 2.0 analysis. This genomic instability profile provides mechanistic insight into the observed tsRNA expression heterogeneity, as CNV-driven gene dosage effects may directly modulate tsRNA biogenesis pathways.

The co-occurrence of 5p15.33 amplifications and 3p deletions across subtypes was particularly interesting, a genomic signature previously associated with epigenetic dysregulation in gastrointestinal malignancies. These findings collectively suggest that tsRNA subtypes encapsulate both transcriptomic and genomic dimensions of gastric cancer heterogeneity.

### 3.4 Mutation pattern differences among tsRNA subtypes

**Figure 4:**
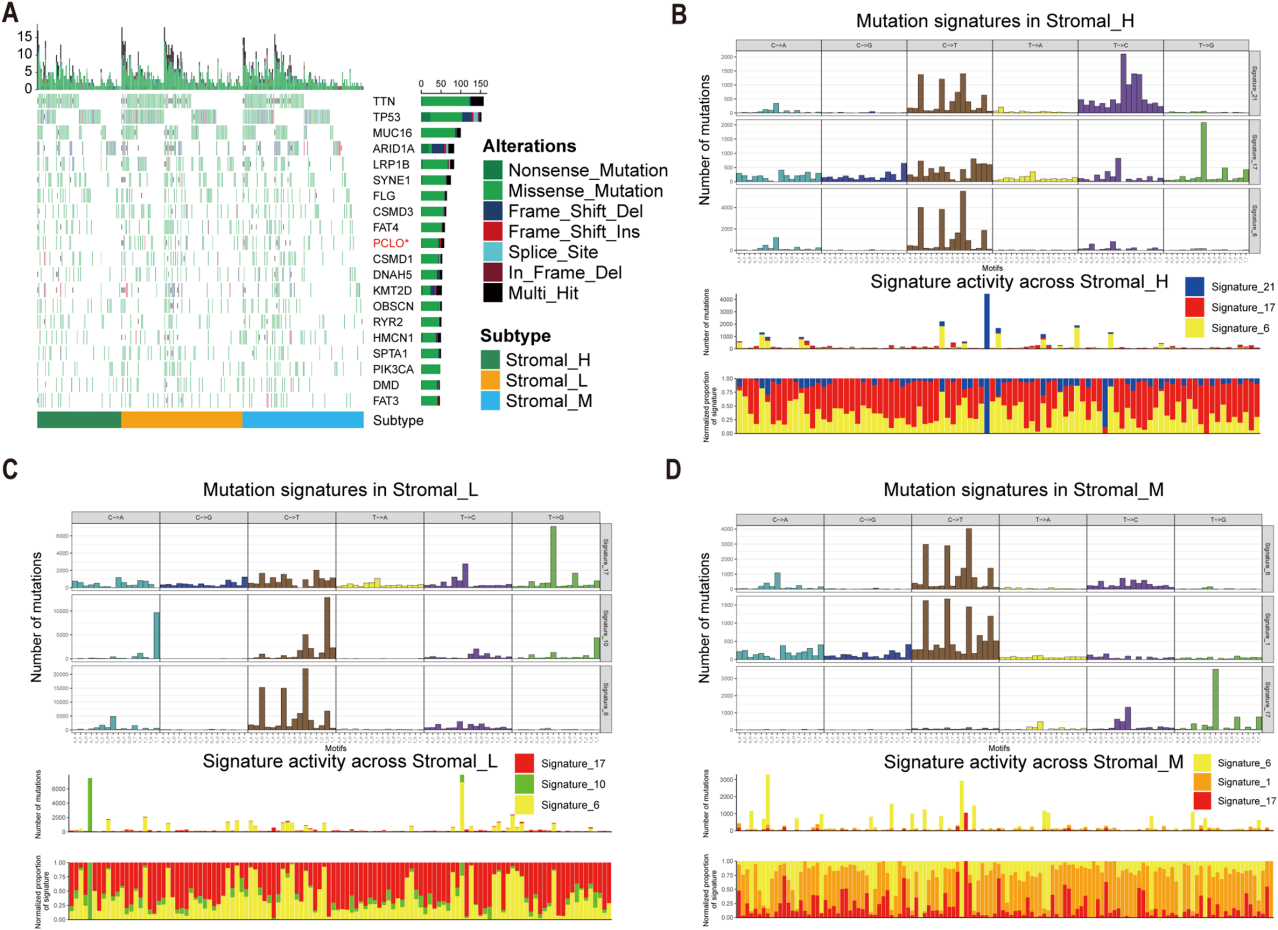
Mutational landscape in tsRNA subtypes. **(A)** Mutational landscape of gene between three tsRNA subtypes. **(B-D)** Mutation patterns in three tsRNA subtypes.

To investigate potential links between the defined tsRNA subtypes and somatic mutation profiles, we performed a significant mutated genes (SMG) analysis across the gastric cancer cohort stratified by subtype. Using waterfall plots, visualizing gene mutation frequencies revealed distinct mutation patterns segregating with the three tsRNA subtypes. Notably, the mutation rate of the PCLO gene exhibited a statistically significant difference among the subtypes (Fisher-test, *P* < 0.05) (Fisher-test, *P* < 0.05) (**Figure 4A**).

To further characterize the underlying mutational processes associated with each tsRNA subtype, we extracted mutational signatures from the whole-exome sequencing data of gastric cancer samples, leveraging the COSMIC mutational signatures database (v2) (https://cancer.sanger.ac.uk/signatures/signatures_v2/). This analysis identified signature 21 as independently associated with the Stromal_H subtype (**Figures 4B-D**). This association suggests a unique mutational etiology specific to this high-stromal group. Furthermore, the mutation profile observed in the Stromal_H group indicated defective DNA mismatch repair, providing a mechanistic insight into the genomic instability features of this subtype.

### 3.5 Development and validation of an RSF-based tsRNA prognostic model

**Figure 5:**
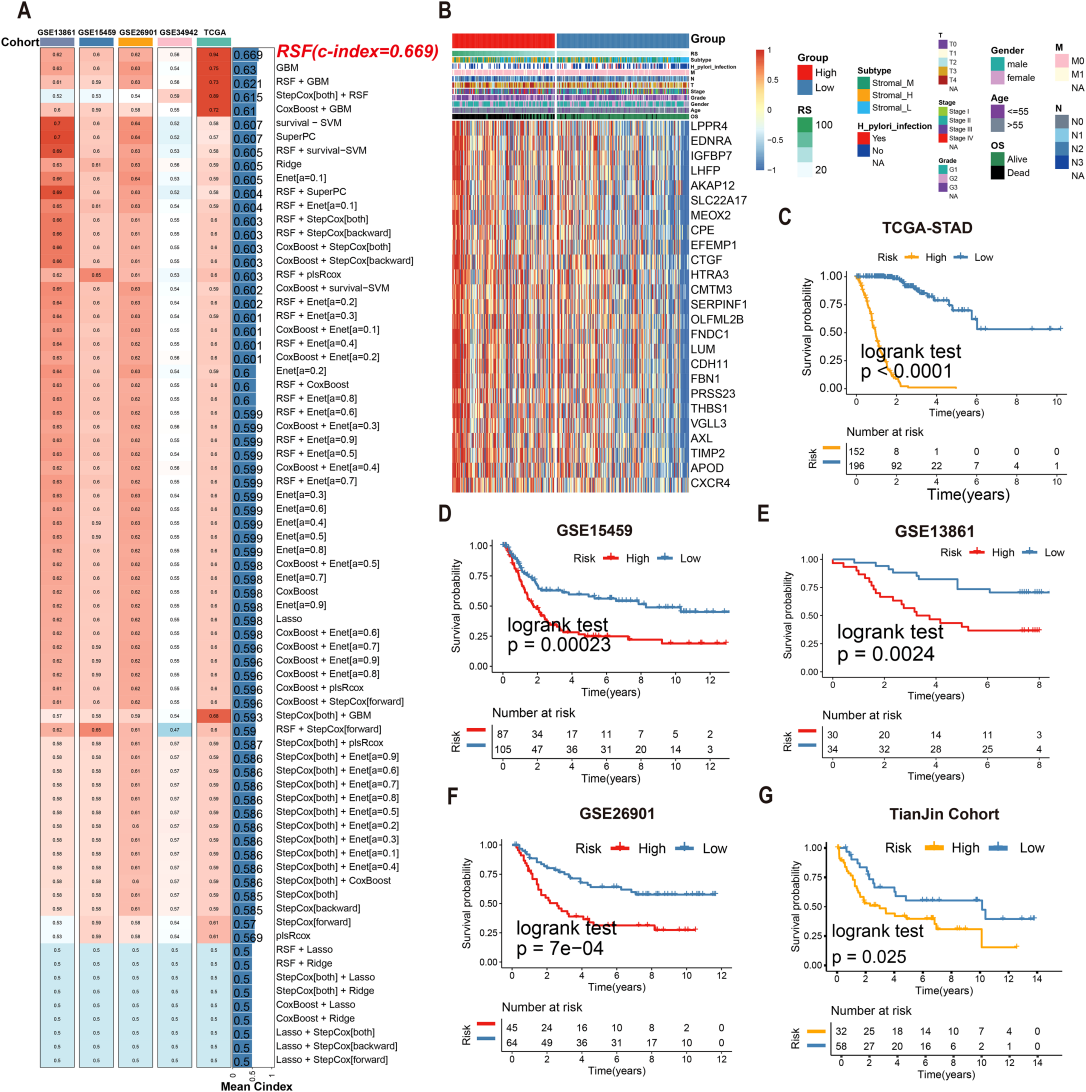
Multiple machine learning algorithms were utilized to construct tsRNA-related prognostic model. **(A)**74 machine learning algorithm combinations were employed to construct prognostic model based on ten machine learning algorithms. **(B, C)** Kaplan-Meier analysis generated recurrence-free survival (RFS) curves for the TCGA cohort **(D)** The expression of 25 mRNAs in GC patients. **(E–G)** Survival analysis of RS in the GEO cohort was created using Kaplan-Meier curves.

**Figure 6:**
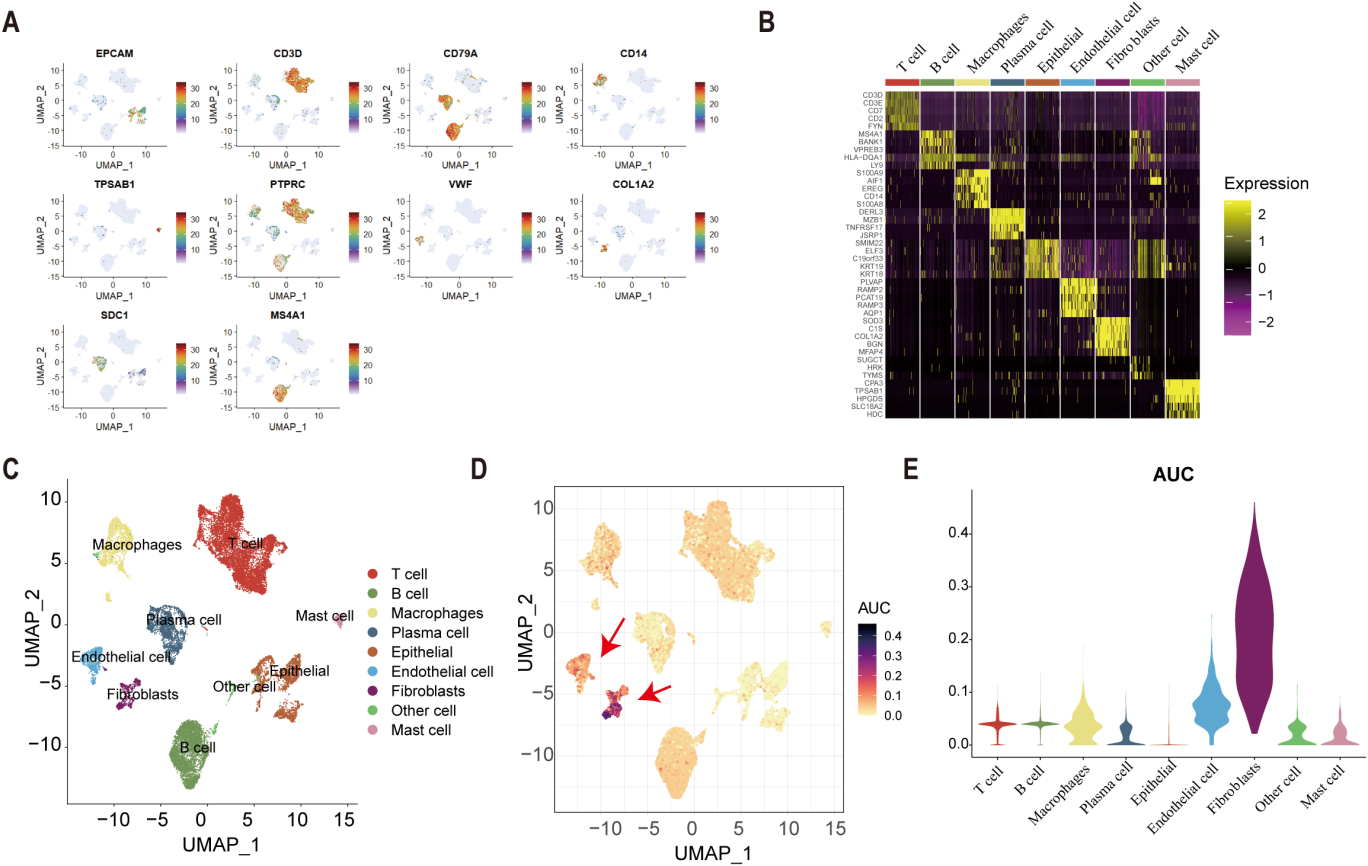
Single-cell analysis. **(A)** cell marker genes. **(B)** The expression of cell marker genes in nine types of cell. **(C)** The result of cell annotation. **(D-E)** The result of AUcell score in nine types of cells.

Univariate Cox regression identified 25 tsRNA-associated mRNAs (*P* < 0.005) from 375 subtype-specific DEmRNAs as having significant survival relevance. Ten machine learning algorithms were systematically evaluated for prognostic model construction. The Random Survival Forest (RSF) algorithm demonstrated optimal performance, achieving the highest concordance index (C-index = 0.669) across all gastric cancer (GC) cohorts (**Figure 5A**). Using the RSF-derived tsRNA score (RS), patients were stratified into high- and low-risk groups based on a predefined cutoff (RS = 44.13084). The low-RS group exhibited significantly superior overall survival compared to the high-RS group (log-rank test, *P* < 0.0001; **Figure 5B**). This prognostic robustness was further validated by receiver operating characteristic (ROC) analysis (5-year AUC = 0.967; **Figure 5C**), with expression patterns of the 25 model genes visualized in a heatmap (**Figure 5D**). External validation using independent GEO cohort and TianJin cohorts confirmed the model’s generalizability, consistently stratifying patients into high- and low-risk groups with distinct survival outcomes (**Figures 5E–G**). Single-cell analysis revealed predominant expression of the 25 model genes in fibroblast and endothelial cell populations (**Figures 6A–E**). To facilitate clinical implementation, we developed the R package “GCtsRNAscore” (https://github.com/huxintmu/GCtsRNAscore) for automated tsRNA prognostic scoring in GC patients (**Supplementary Figure S5**).

### 3.6 Clinical utility of the tsRNA score in treatment stratification

**Figure 7:**
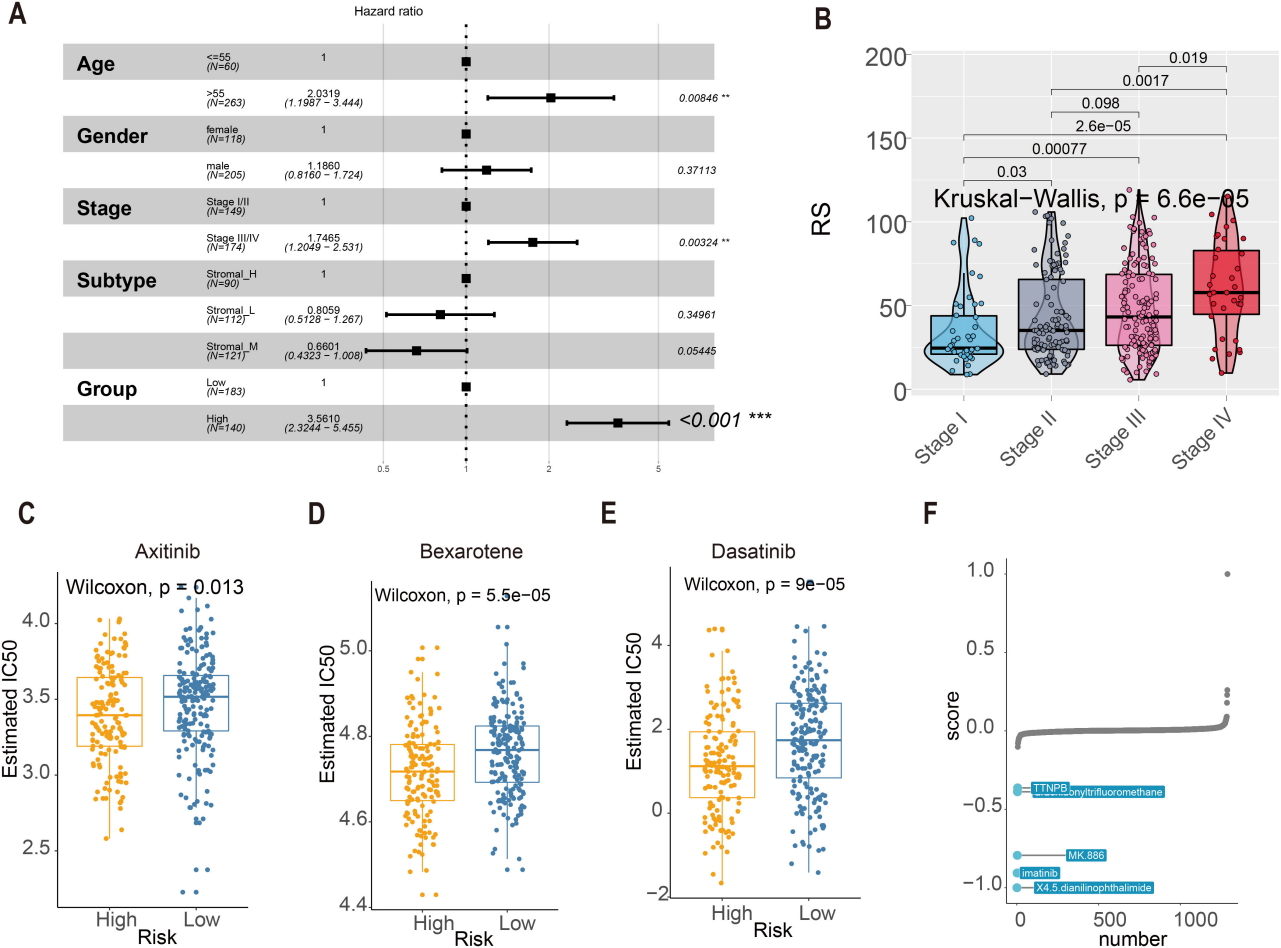
RS score and clinical indicators. **(A)** Multivariate Cox regression assessed associations between clinicopathological variables and overall survival (OS) in the TCGA cohort. **(B)** The boxplot showed RS was elevated as the tumor stage progressed (Kruskal-Wallis statistical test, *P* = 6.6e-05). **(C–E)** The IC50 values of three chemotherapeutic agents with RS. Axitinib, Bexarotene, and Dasatinib. **(F)** Top 5 Small-molecule compounds with RS.

Multivariate Cox regression confirmed the RS as an independent prognostic factor independent of clinicopathological variables (age, gender, stage; **Figure 7A**). Notably, RS values increased significantly with advancing tumor stage (Kruskal–Wallis test, *P* = 6.6e-05; **Figure 7B**), underscoring its clinical relevance. For therapeutic guidance, chemotherapy response analysis using the R package “pRRophetic” revealed distinct drug sensitivities between risk groups: In silico drug sensitivity analysis using the pRRophetic algorithm suggested that the high-RS group may exhibit potentially enhanced sensitivity to axitinib, bexarotene, and dasatinib (**Figures 7C–E**). These predictive findings provide a potential basis for further experimental and clinical validation and may guide future mechanistic and translational studies. Complementary analysis via the eXtreme Sum (XSum) algorithm further identified X4.5.dianilinophthalimide as a potential therapeutic candidate for high-RS patients (**Figure 7F**).

**Figure 8:**
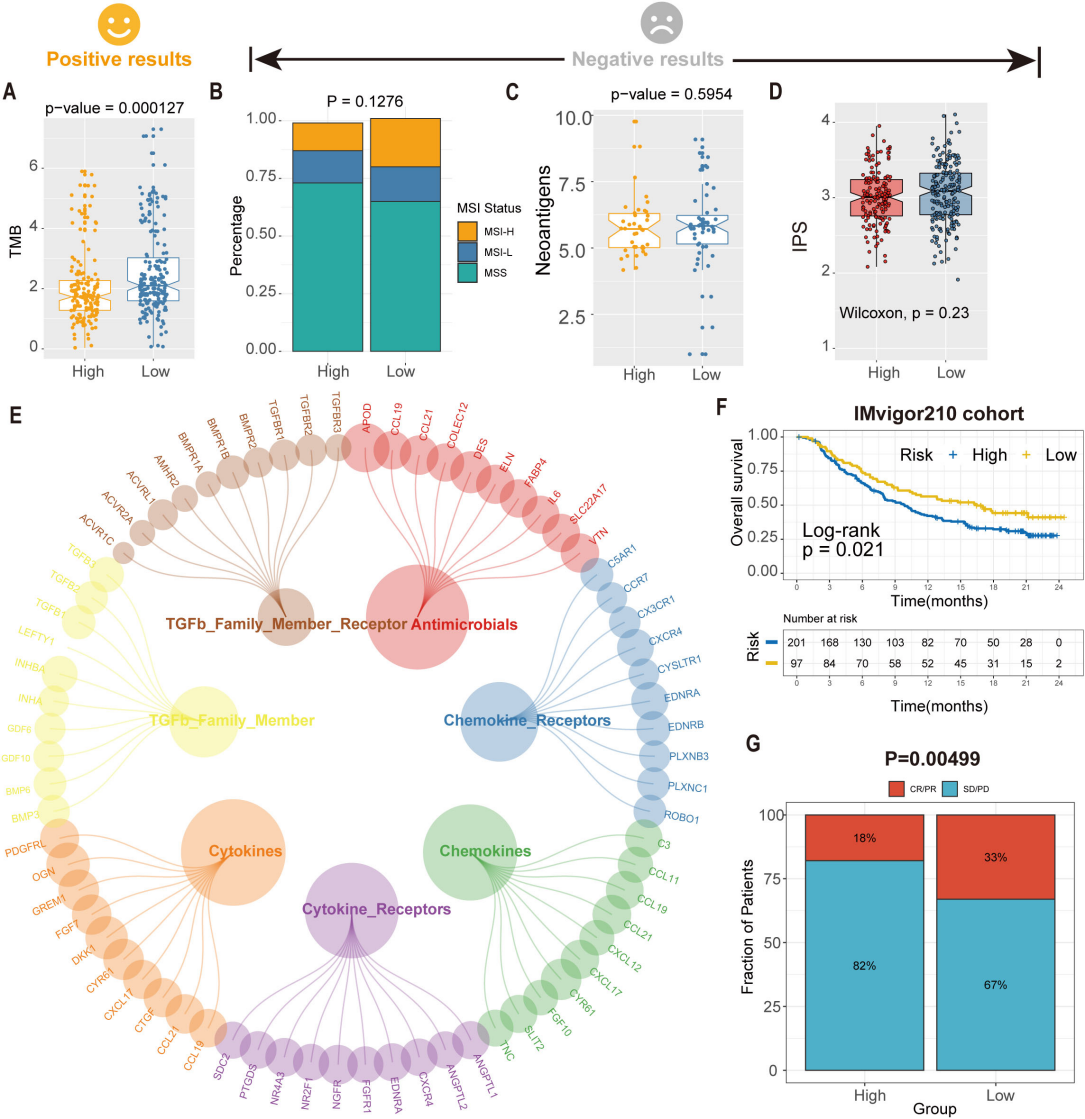
Predicted tsRNA associated with immunotherapy response and outcomes in GC. **(A)**The boxplot showed a statistically different TMB between high- and low-RS groups. **(B)** The proportion of MSI status in high versus low RS subgroups. **(C)** The boxplot showed a statistical difference in neoantigens between high- and low-RS groups. **(D)** The boxplot showed no statistical difference in IPS between high- and low-RS groups. **(E)** The correlation of RS and immune-related pathway. **(F)** Kaplan-Meier analysis of the high versus low RS subgroup in the IMvigor210 cohort. **(G)** The proportion of immune response to immunotherapy in high versus low RS subgroups. CR, complete response; PR, partial response; SD, stable disease; PD, progressive disease.

To evaluate the predictive capacity of the risk score (RS) model in immunotherapy response, we conducted comprehensive analyses of tumor mutational burden (TMB) and immune-related biomarkers. TMB analysis revealed significantly divergent responses to immunotherapy between high- and low-RS groups (*P* < 0.05; **Figure 8A**). In contrast, microsatellite instability (MSI) status, immunophenoscore (IPS), and neoantigen load exhibited no significant differences between the subgroups (*P* > 0.05; **Figures 8B–D**). These findings suggest that RS-stratified subgroups may exhibit differential sensitivity to immunotherapy independent of conventional biomarkers.

Further investigation identified seven immune-related pathways (e.g., antigen processing, cytokine signaling) that were differentially activated between high- and low-RS groups (Wilcoxon test, *P* < 0.05; **Figure 8E**), providing mechanistic insights into the observed therapeutic heterogeneity. We analyzed the IMvigor210 cohort (anti-PD-L1-treated bladder cancer patients) to validate clinical relevance using the RS model. Kaplan-Meier analysis demonstrated significantly prolonged overall survival (OS) in low-RS patients compared to high-RS counterparts (log-rank *P* = 0.021; **Figure 8F**). Critically, low-RS patients exhibited a 2.3-fold higher objective response rate (ORR; complete response [CR] + partial response [PR]) to immunotherapy than high-RS patients (35.2% vs. 15.4%, *P* = 0.00499; **Figure 8G**), highlighting RS as a robust predictor of immunotherapeutic benefit. These findings suggest an association between the RS model and immunotherapy benefit, which warrants further validation in prospective clinical studies.

### 3.7 Validation of bioinformatics analysis results through Pandora sequencing

**Figure 9:**
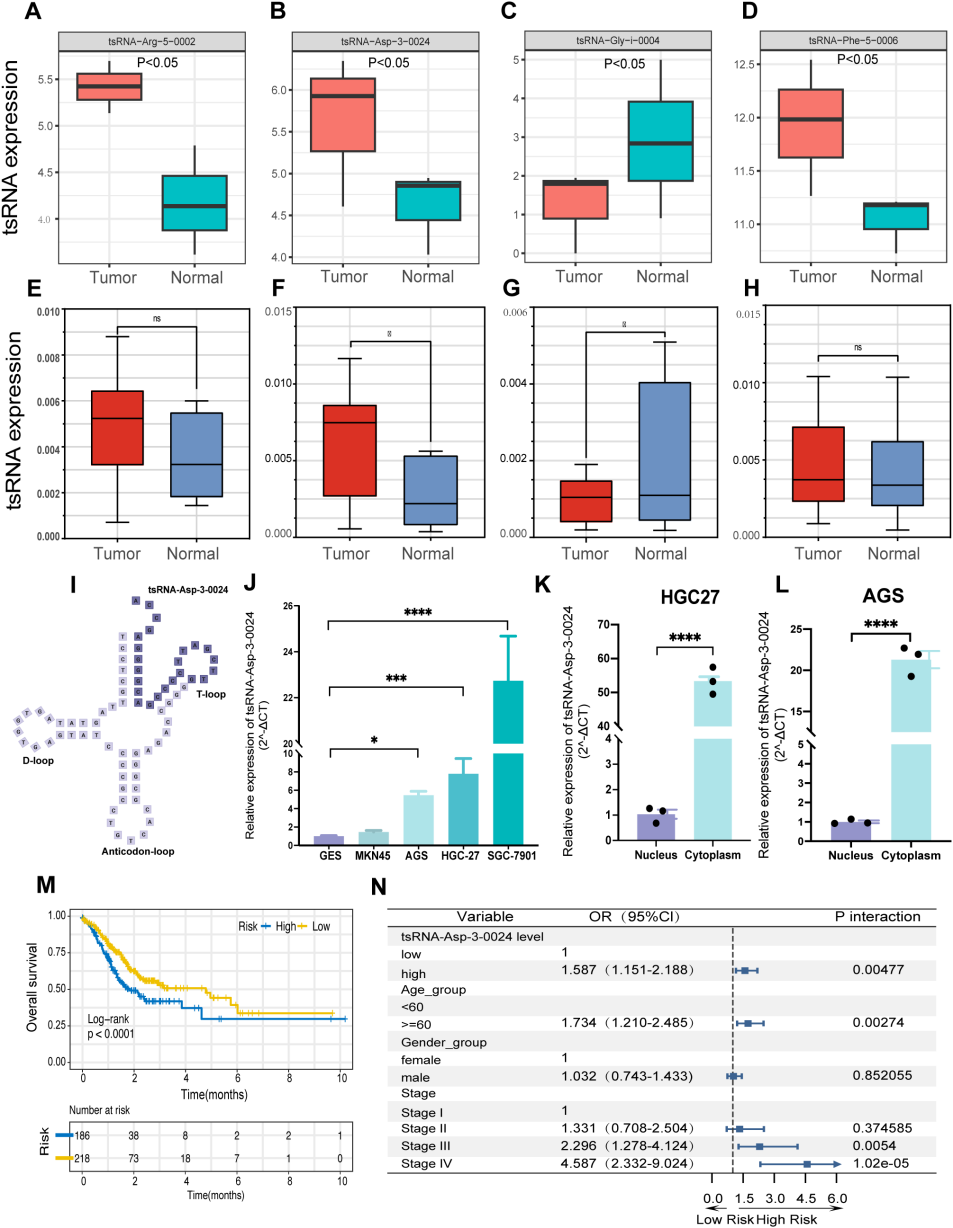
tsRNA-Asp-3–0024 tsRNA-Asp-3–0024 is upregulated in STAD and is significantly associated with poor prognosis.**(A-D)** tsRNA expression in Pandora sequencing. **(D–H)** tsRNA expression in gastric tissues. **(I)** Location of tsRNA-Asp-3–0024 in tRNA-derived fragments. **(J)** The expression levels of tsRNA-Asp-3–0024 in STAD cells were higher than that in normal bronchial epithelial cells (GES). **(K-L)** higher cytoplasmic than nuclear abundance of tsRNA-Asp-3-0024. **(M)** Higher tsRNA-Asp-3–0024 levels were associated with shorter overall survival in STAD patients. **(N)** tsRNA-Asp-3–0024 as an independent prognostic factor in gastric cancer. *p < 0.05, ***p < 0.001, ****p < 0.0001,ns: non-significant.

We performed Pandora sequencing on three pairs of gastric cancer and adjacent tissues to validate the bioinformatics analysis results, identifying 1,300 differentially expressed tsRNAs. Further comparison of tsRNA base pairs confirmed that the expression patterns of six tsRNAs were consistent with the preliminary analysis results (**Figures 9A-D**).

### 3.8 tsRNA-Asp-3–0024 is upregulated in GC and is significantly associated with poor prognosis

Based on the results of the previous analysis, we further collected 10 pairs of gastric cancer and adjacent tissue samples for qRT-PCR validation to detect the expression levels of the four tsRNAs obtained (**Supplementary Table S4**). Expression levels of tsRNA-Phe-5–0006 and tsRNA-Arg-5–0002 showed no significant difference between cancerous and adjacent tissues (**Figures 9E, H**). In contrast, tsRNA-Asp-3–0024 was significantly upregulated in tumor tissue (**Figure 9F**), whereas tsRNA-Gly-i-0004 was significantly downregulated (**Figure 9G**), suggesting potential involvement of both tsRNAs in gastric tumorigenesis. Notably, tsRNA-Asp-3–0024 represents a novel, previously unreported tRF. Derived from nucleotides 52–75 at the 5ʹ-end of tRNA-Asp-GTC-2-10, it is a 24-nt 3ʹ-tRF (**Figure 9I**). qRT-PCR confirmed its significant upregulation in gastric cancer tissue (**Figure 9F**). Consistently, tsRNA-Asp-3–0024 expression was elevated in STAD cell lines (HGC27, MKN45, AGS, HGC7901) compared to normal gastric epithelial cells (**Figure 9J**). Subcellular fractionation revealed higher cytoplasmic than nuclear abundance of tsRNA-Asp-3-0024 (**Figures 9K, L**). We integrated TCGA-STAD clinical data with tsRFun-derived expression profiles for prognostic assessment to evaluate its clinical relevance. Clinical analysis demonstrated that elevated tsRNA-Asp-3–0024 was associated with shorter overall survival in GC patients (log-rank *P* < 0.0001) and functioned as an independent prognostic factor by multivariable Cox regression (**Figures 9M, N**).

### 3.9 Knockdown of tsRNA-Asp-3–0024 inhibits proliferation in GC cells and organoids

**Figure 10:**
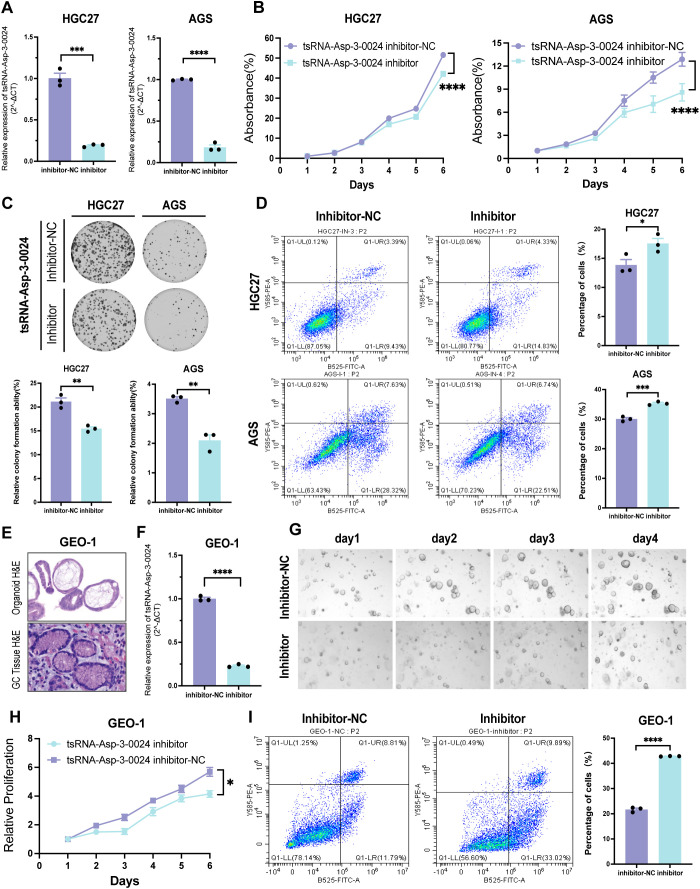
Knockdown of tsRNA-Asp-3–0024 inhibits proliferation in STAD cells and organoids **(A)** Transfection efficiency of tsRNA-Asp-3–0024 inhibitor in gastric cancer cell lines detected by qRT-PCR. **(B, C)** tsRNA-Asp-3–0024 knockdown significantly inhibited the proliferation of HGC-27 and AGS cells. **(D)** Flow cytometry analysis showed that knockdown significantly increased apoptosis rates in HGC-27 and AGS cells. **(E)** HE staining for organoid identification. **(F)** Transfection efficiency of tsRNA-Asp-3–0024 mimics in gastric organoid detected by qRT-PCR. **(G)** Organoids grow on the fourth day after transfection of organoids. **(H)** GC organoids’ growth is summarized using a line chart. **(I)** Flow cytometry analysis revealed that knockdown promoted organoid apoptosis. *p < 0.05, ***p < 0.001, ****p < 0.0001.

To assess the biological function of tsRNA-Asp-3–0024 in GC, we established tsRNA-Asp-3–0024 knockdown models in HGC-27 and AGS cells. Knockdown efficiency was confirmed by qRT-PCR in both cell lines (**Figure 10A**). CCK-8 assays demonstrated that tsRNA-Asp-3–0024 knockdown significantly inhibited proliferation in HGC-27 and AGS cells (**Figure 10B**). Colony formation assays further revealed that knockdown markedly reduced colony formation in these cells (**Figure 10C**). Flow cytometry analysis showed that knockdown significantly increased apoptosis rates in HGC-27 and AGS cells compared to controls (**Figure 10D**). To better model the tumor immune microenvironment *in vivo*, we validated these findings using patient-derived gastric cancer organoids. Histopathological assessment of H&E-stained organoids confirmed they recapitulated gastric cancer features consistent with the original patient pathology (**Figure 10E**). Following successful establishment of tsRNA-Asp-3–0024 knockdown in organoids (**Figure 10F**), 3D viability assays demonstrated that knockdown significantly suppressed organoid proliferation (**Figures 10G, H**). Flow cytometry analysis revealed that knockdown promoted organoid apoptosis (**Figure 10I**), consistent with cellular findings. These results demonstrate that tsRNA-Asp-3–0024 knockdown exerts tumor-suppressive effects on gastric cancer progression. In addition, we have conducted additional analyses using the ssGSEA algorithm to calculate the tsRNA-Asp-3–0024 score in 15 gastric cancer cohorts (**Supplementary Figure S6A**). Our analysis revealed that the tsRNA-Asp-3–0024 score activates several cancer-related pathways, including Mtorc1 signaling, Myc targets v1, DNA replication, and Cell cycle (**Supplementary Figure S6B–D**). These findings suggest that tsRNA-Asp-3–0024 may exert its tumor-promoting effects through the regulation of these key pathways.

## 4 Discussion

Gastric cancer is a highly heterogeneous malignancy, with survival outcomes varying dramatically by stage and molecular characteristics (36, 37). Historically, clinical classification systems such as those by Lauren, Nakamura, and the WHO have been foundational for diagnosis and treatment decisions, but these frameworks often lack the granularity needed to capture the full spectrum of molecular diversity in GC (38–40). Recent genomic studies-including those by TCGA and ACRG-have further delineated GC subtypes based on genetic, epigenetic, and transcriptional landscapes, revealing distinct biological and clinical features (36, 37). However, these classifications are rarely translated into routine clinical practice, and reliable biomarkers for early detection and prognosis remain urgently needed. We found that our subtypes show distinct characteristics compared to the TCGA (P = 0.004027) and ACRG (P = 6.19e-09) classifications (**Supplementary Figure S3 A, B**). This analysis highlights the unique aspects of our classification. Compared to the ACRG subtypes, the EMT subtype has the highest proportion in the stromal-H subtype and the worst prognosis, while the stromal-L subtype has the best prognosis and does not include the EMT subtype. Compared with TCGA subtypes, stromal-H had higher proportions of CIN and GS subtypes, indicating a poorer prognosis, while the MSI subtype was most prevalent in stromal-L.

Our study demonstrates that tsRNA expression signatures can robustly define three molecular subtypes of gastric cancer, each associated with unique tumor microenvironment profiles and clinical outcomes. The Stromal_H subtype was linked to higher stromal infiltration, a greater frequency of DNA repair gene mutations, and poorer prognosis, while Stromal_L was associated with more favorable outcomes. These findings are consistent with prior research showing that stromal and immune cell composition significantly influence tumor progression and therapeutic response in GC (41–44).

We further developed and validated a tsRNA-based prognostic model using machine learning algorithms. This model accurately stratified patients into high- and low-risk groups, correlating with both survival outcomes and therapeutic response. High-risk patients identified by the tsRNA risk score were more likely to benefit from targeted therapies such as axitinib, bexarotene, and dasatinib. In contrast, low-risk patients had better responses to immunotherapy. These results highlight the potential of tsRNA profiling to guide precision medicine in gastric cancer.

It is important to note that drug sensitivity predictions (e.g., axitinib, dasatinib) and immunotherapy response analyses (e.g., TMB, IPS, IMvigor210) presented in this study are based on computational inference using publicly available algorithms and datasets. These findings should be considered exploratory and do not constitute direct evidence of clinical efficacy. Prospective clinical trials and *in vitro*/*in vivo* validation are essential to confirm the predictive value of the RS model before it can be considered for guiding treatment decisions.

**Figure 11:**
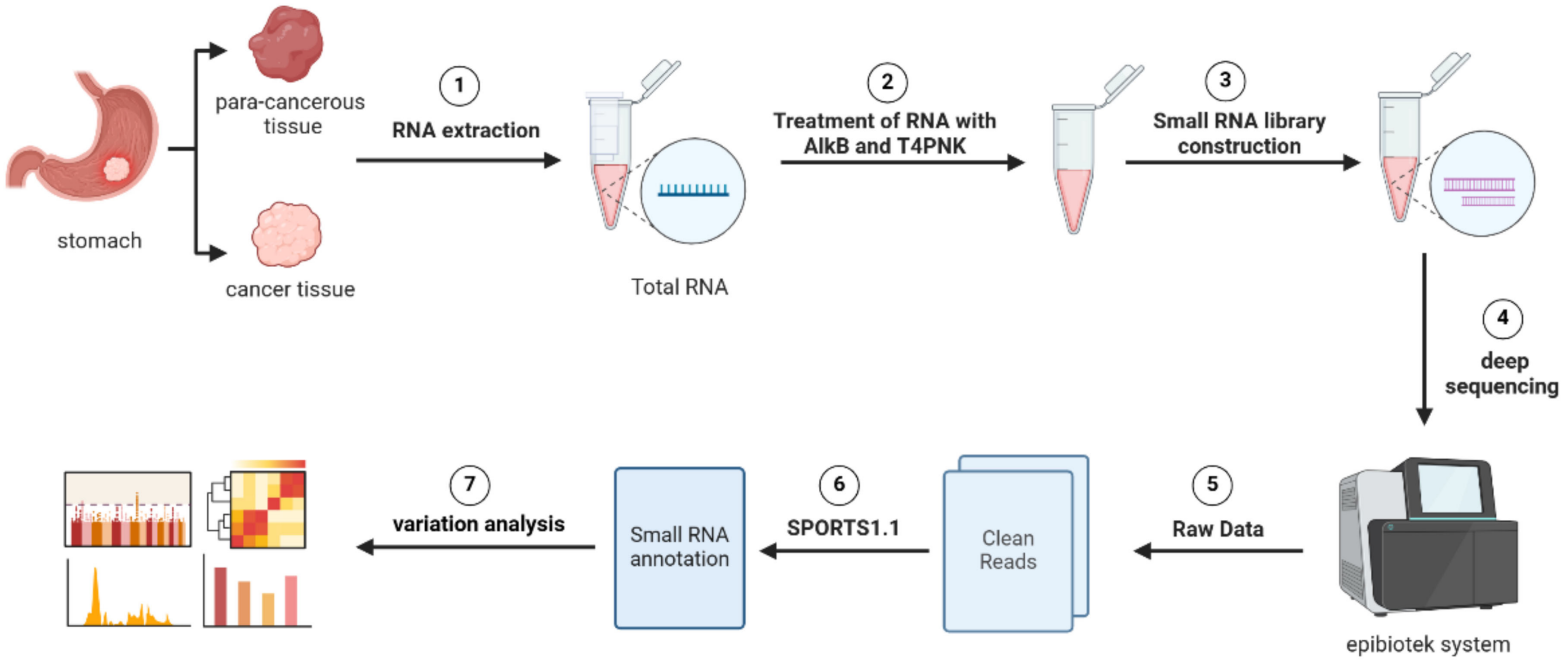
Workflow of Pandora-seq for comprehensive tsRNA profiling. The schematic illustrates the key experimental and bioinformatic steps. The process begins with RNA extraction from paired gastric cancer and para-cancerous tissues, followed by AlkB and T4 PNK treatment to overcome RNA modification barriers. Subsequent steps include small RNA library construction, deep sequencing, and bioinformatic processing using the epiblotek system and SPORTS1.1 pipeline to generate clean reads and perform variation analysis.

Importantly, we identified tsRNAAsp-3–0024 as a novel independent prognostic biomarker through Pandora sequencing and validated it experimentally (**Figure 11**). Elevated expression of tsRNA-Asp-3–0024 was consistently linked to poor survival in GC patients and promoted tumor cell proliferation in both cellular and organoid models. Knockdown of tsRNA-Asp-3–0024 suppressed tumor growth and increased apoptosis, suggesting a functional role in GC pathogenesis. Previous studies have similarly shown that tRNA-derived fragments can regulate oncogenic pathways and influence tumor biology (45–48), supporting our findings.

Our multi-cohort analysis reveals that the tsRNA-Asp-3–0024 score is significantly associated with the activation of key oncogenic pathways, including mTORC1 signaling, Myc targets, DNA replication, and the Cell cycle. This strongly suggests that tsRNA-Asp-3–0024 contributes to gastric cancer progression by coordinately enhancing tumor-promoting signals. Given that mTORC1 and Myc are well-established central regulators of cell growth and proliferation (49, 50), their concurrent enrichment implies that tsRNA-Asp-3–0024 may function as a high-level regulator. As tsRNAs can post-transcriptionally regulate gene expression (51), we hypothesize it may promote proliferation by repressing tumor suppressors or stabilizing mRNAs of cell cycle proteins, a mechanism documented for other tsRNAs (52). Thus, tsRNA-Asp-3–0024 emerges as a potential key player and therapeutic target in gastric cancer.

Our results also provide new insight into the relationship between tsRNAs, fibroblasts, and the tumor microenvironment. Single-cell analysis indicated that key tsRNA-related genes were highly expressed in fibroblast and endothelial populations, which are increasingly recognized as drivers of GC aggressiveness and therapeutic resistance (41–43).

While this study leveraged comprehensive multi-omics approaches and rigorous experimental validation, several limitations remain. Our analyses were primarily based on retrospective cohorts, and future prospective studies will be required to confirm the clinical utility of tsRNA-based biomarkers and models in diverse patient populations. Additional mechanistic studies are warranted to elucidate the regulatory networks connecting tsRNA expression to tumor progression and immune response.

Our work establishes tsRNAs as robust molecular markers for gastric cancer subtyping and prognosis. TsRNA-Asp-3-0024, in particular, emerges as a promising biomarker and therapeutic target. Integrating tsRNA profiling into clinical workflows may significantly enhance risk stratification, early detection, and personalized treatment in gastric cancer.

## Data Availability

The original contributions presented in the study are included in the article/**supplementary material**. Further inquiries can be directed to the corresponding authors.

## Supplementary material

Supplementary Figure 1 The workflow of our study.

Supplementary Figure 2 Kaplan-Meier (K-M) survival analysis in the supplementary data set.

Supplementary Figure 3 tsRNA subtypes exhibit significantly different characteristics in TCGA and ACRG.

Supplementary Figure 4 375 differentially expressed mRNAs (DEmRNAs) in three tsRNA subtypes. (cutoff thresholds:P < 0.05 and |log2 fold change| > 0.5).

Supplementary Figure 5 The computational flow of R package “ GCtsRNAscore”.

Supplementary Figure 6 ssGSEA algorithm analysis of 15 gastric cancer cohorts.

Supplementary Table 1 The expression of GC-specific 80 tsRNAs in TCGA.

Supplementary Table 2 75 differentially expressed mRNAs were determined using the cutoff thresholds of *P* < 0.05 and |log2 fold change| > 0.5.

Supplementary Table 3 Protein-protein interaction (PPI) network analysis showed 67 hub genes in 375 DEmRNAs (node degree > 20).

Supplementary Table 4 The clinical characteristics of GC samples for the Pandora-seq, qRT-PCR, and CISH analysis.
