# Supplementary figures and images for: Functional and clinical validation of tsRNA-defined molecular subtypes guides precision therapy in gastric cancer
Source: Front Immunol. 2025 Nov 3;16:1684113. doi: 10.3389/fimmu.2025.1684113 (PMC12620406; doi:10.3389/fimmu.2025.1684113)

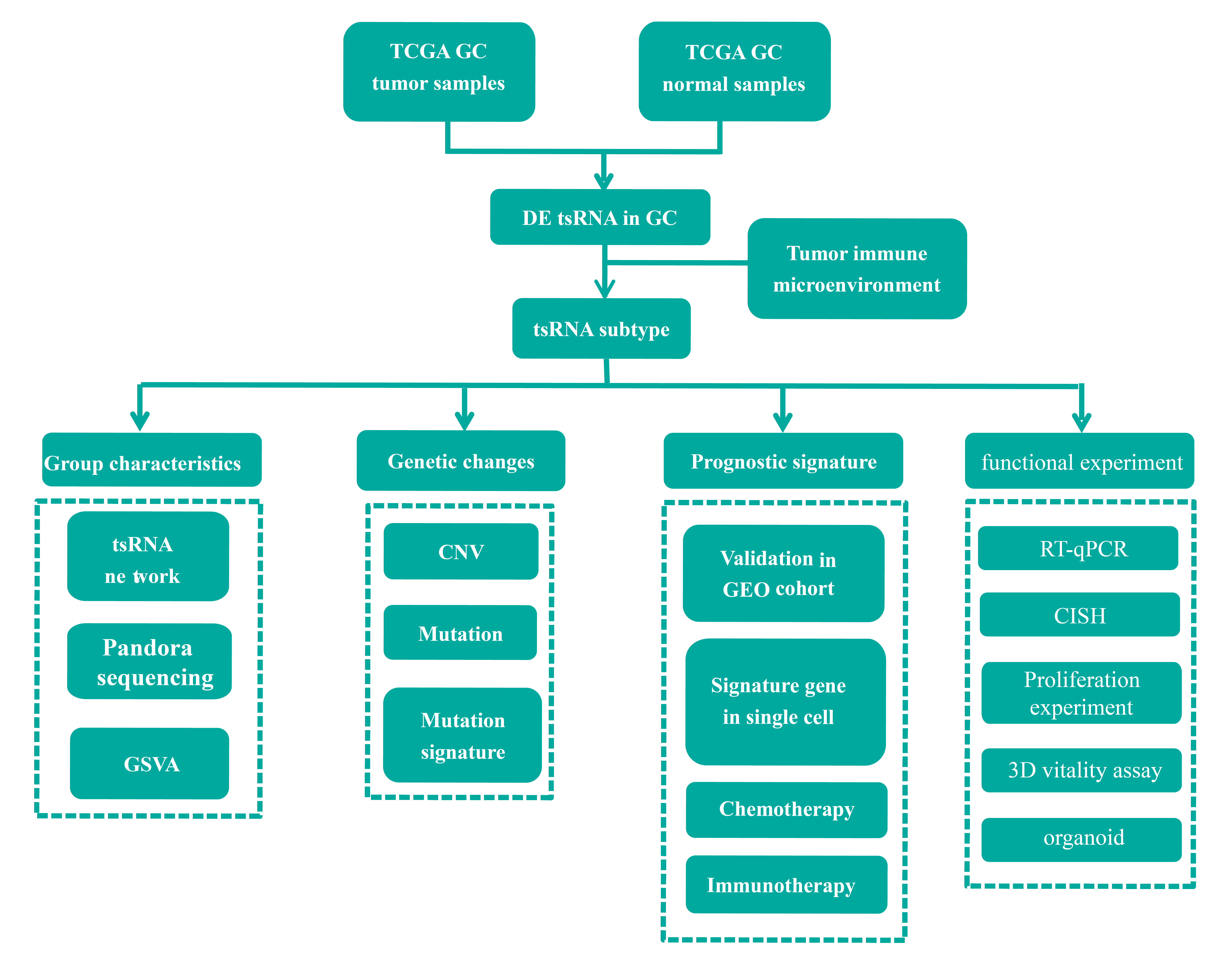

Supplement: Supplementary Figure 1 — The workflow of our study. [file Image1.tif]

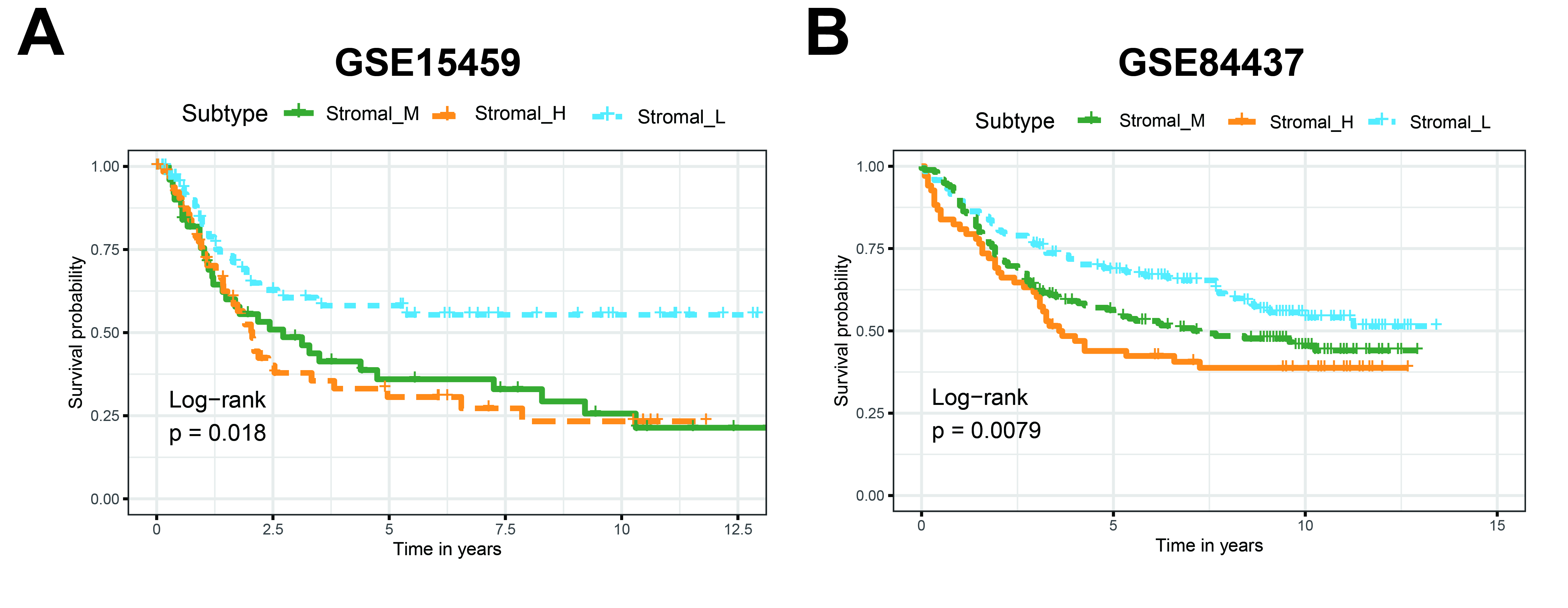

Supplement: Supplementary Figure 2 — Kaplan-Meier (K-M) survival analysis in the supplementary data set. [file Image2.tif]

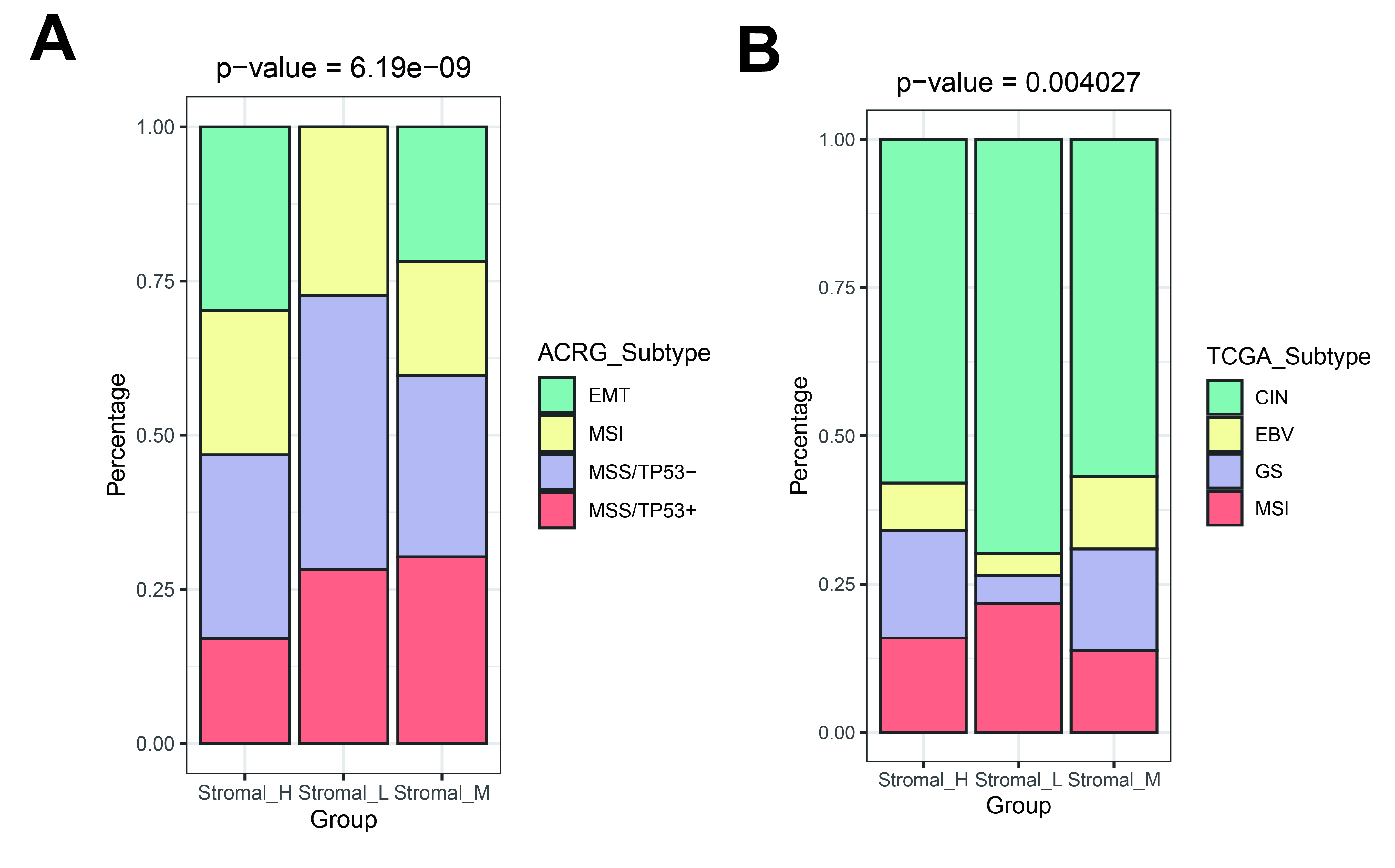

Supplement: Supplementary Figure 3 — tsRNA subtypes exhibit significantly different characteristics in TCGA and ACRG. [file Image3.tif]

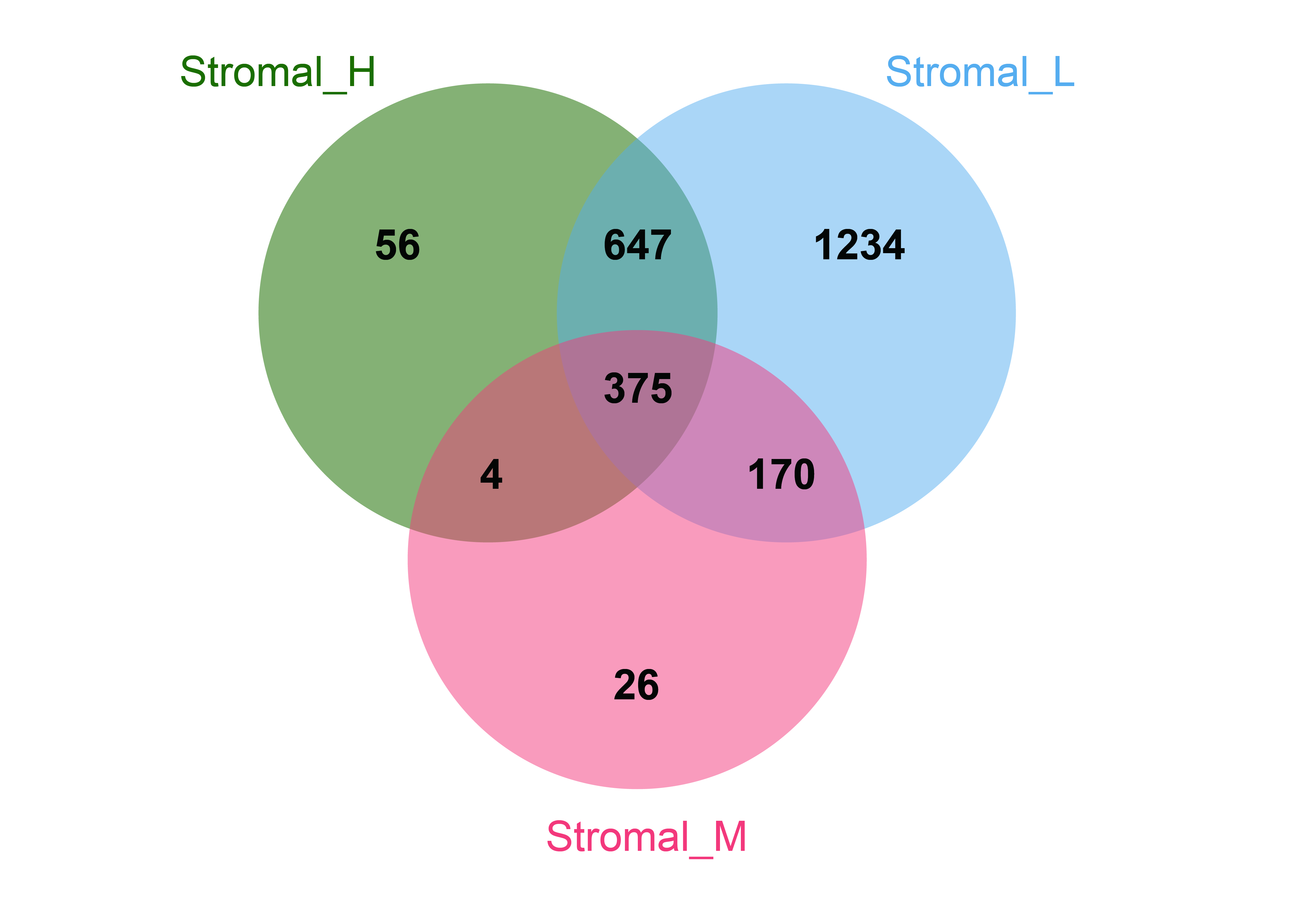

Supplement: Supplementary Figure 4 — 375 differentially expressed mRNAs (DEmRNAs) in three tsRNA subtypes. (cutoff thresholds:P < 0.05 and |log2 fold change| > 0.5). [file Image4.tif]

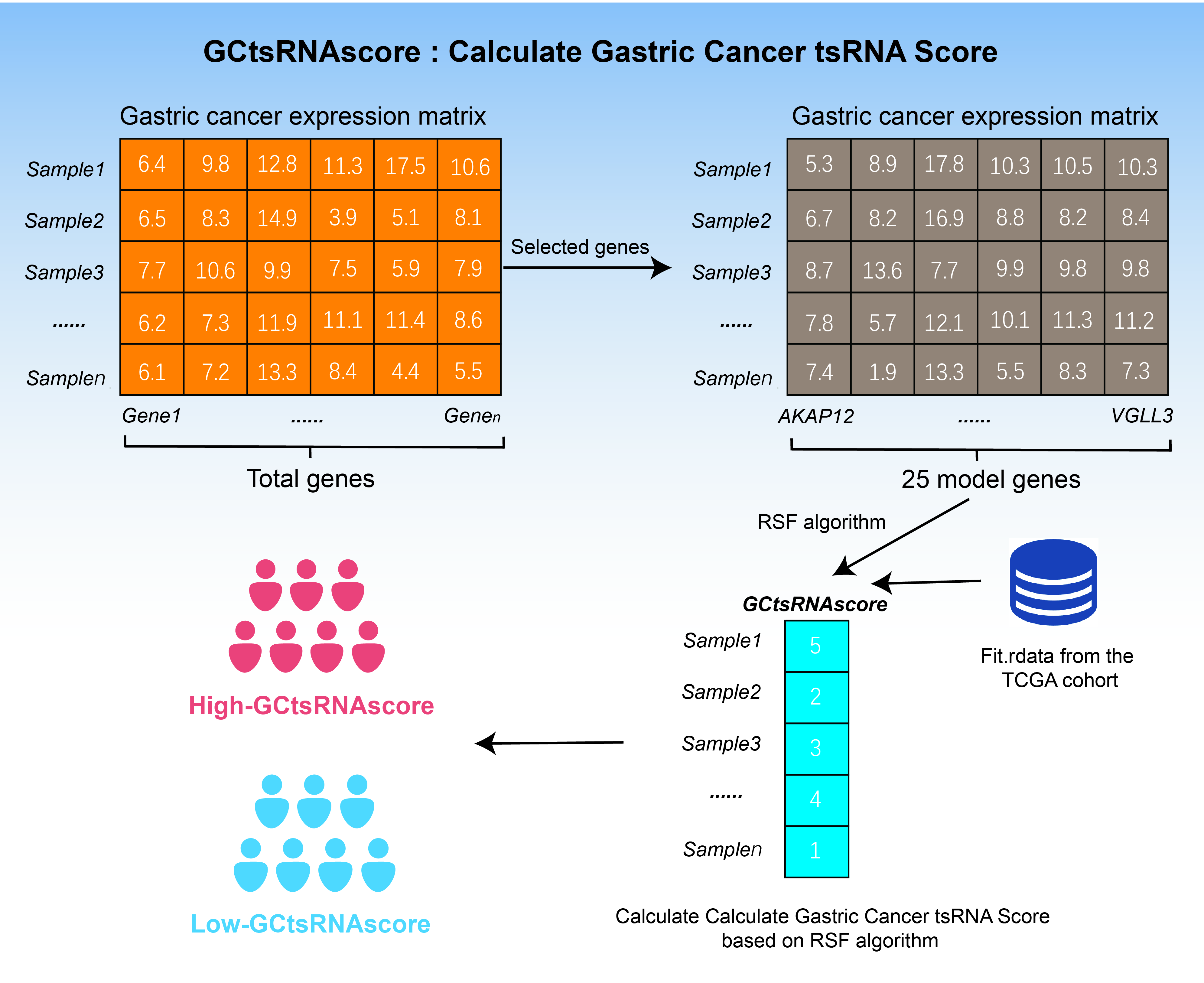

Supplement: Supplementary Figure 5 — The computational flow of R package “ GCtsRNAscore”. [file Image5.tif]

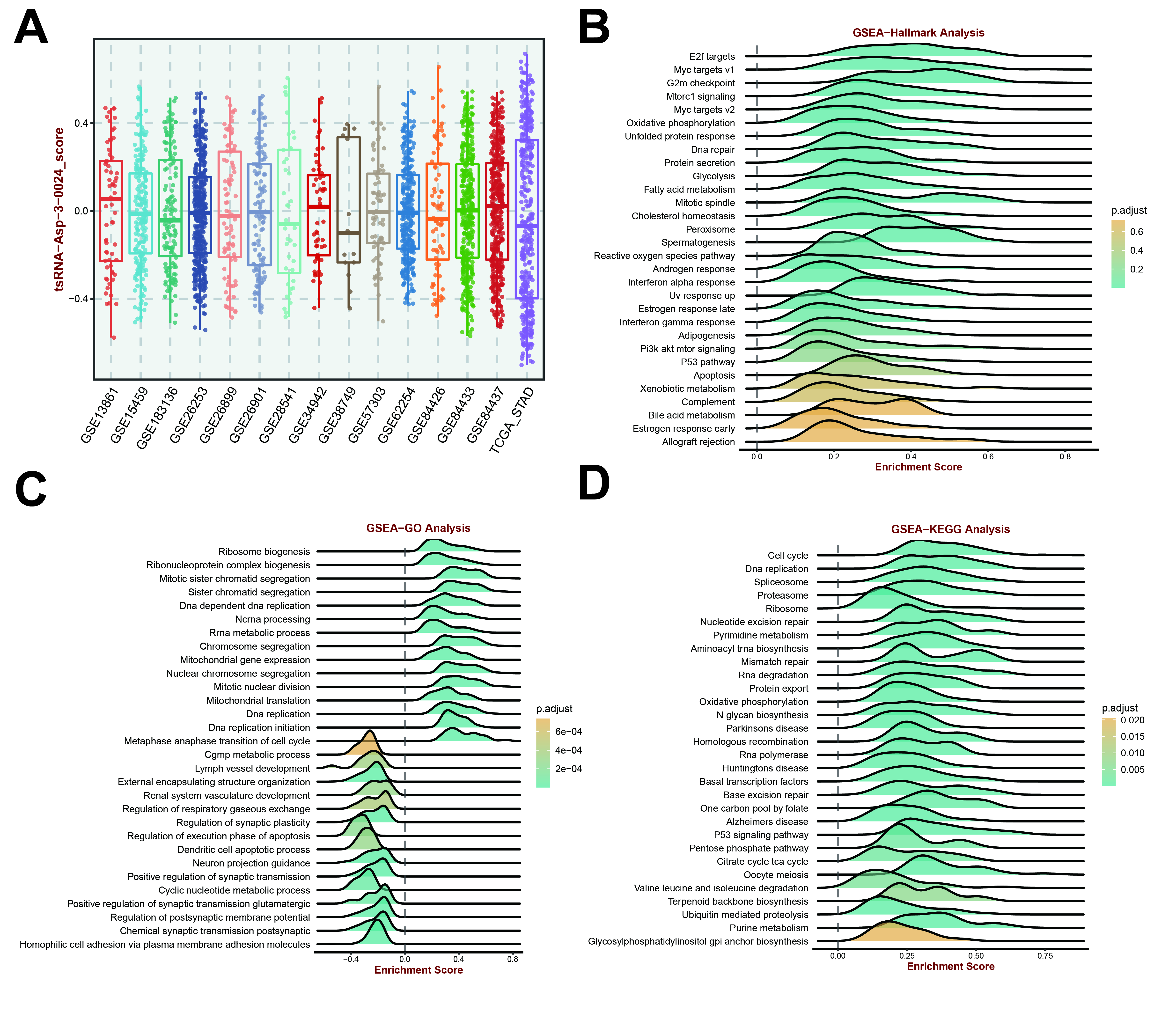

Supplement: Supplementary Figure 6 — ssGSEA algorithm analysis of 15 gastric cancer cohorts. [file Image6.tif]
